# Hepatocyte polarity establishment and apical lumen formation are organized by Par3, Cdc42, and aPKC in conjunction with Lgl

**DOI:** 10.1016/j.jbc.2021.101354

**Published:** 2021-10-27

**Authors:** Vlad Tocan, Junya Hayase, Sachiko Kamakura, Akira Kohda, Shouichi Ohga, Motoyuki Kohjima, Hideki Sumimoto

**Affiliations:** 1 Department of Biochemistry, Kyushu University Graduate School of Medical Sciences, Fukuoka, Japan; 2 Department of Pediatrics, Kyushu University Graduate School of Medical Sciences, Fukuoka, Japan; 3 Department of Medicine and Regulatory Science, Kyushu University Graduate School of Medical Sciences, Fukuoka, Japan

**Keywords:** atypical protein kinase C, Cdc42, cell polarity, hepatocyte, Lgl, Par3, aPKC, atypical protein kinase C, BC, bile canaliculus, BSA, bovine serum albumin, CCR, C-terminal conserved region, cDNA, complementary DNA, DPP4, dipeptidyl peptidase IV, ECM, extracellular matrix, FBS, fetal bovine serum, HEK293T, human embryonic kidney 293T, MDCK, Madin–Darby canine kidney, MEM, minimum essential medium, MRP2, multidrug resistance protein 2, NA, numerical aperture, PBR, polybasic region, PM, plasma membrane, SIM, structured illumination microscopy, TJ, tight junction

## Abstract

Hepatocytes differ from columnar epithelial cells by their multipolar organization, which follows the initial formation of central lumen-sharing clusters of polarized cells as observed during liver development and regeneration. The molecular mechanism for hepatocyte polarity establishment, however, has been comparatively less studied than those for other epithelial cell types. Here, we show that the tight junction protein Par3 organizes hepatocyte polarization *via* cooperating with the small GTPase Cdc42 to target atypical protein kinase C (aPKC) to a cortical site near the center of cell–cell contacts. In 3D Matrigel culture of human hepatocytic HepG2 cells, which mimics a process of liver development and regeneration, depletion of Par3, Cdc42, or aPKC results in an impaired establishment of apicobasolateral polarity and a loss of subsequent apical lumen formation. The aPKC activity is also required for bile canalicular (apical) elongation in mouse primary hepatocytes. The lateral membrane-associated proteins Lgl1 and Lgl2, major substrates of aPKC, seem to be dispensable for hepatocyte polarity establishment because Lgl-depleted HepG2 cells are able to form a single apical lumen in 3D culture. On the other hand, Lgl depletion leads to lateral invasion of aPKC, and overexpression of Lgl1 or Lgl2 prevents apical lumen formation, indicating that they maintain proper lateral integrity. Thus, hepatocyte polarity establishment and apical lumen formation are organized by Par3, Cdc42, and aPKC; Par3 cooperates with Cdc42 to recruit aPKC, which plays a crucial role in apical membrane development and regulation of the lateral maintainer Lgl.

Hepatocytes, the specialized epithelial cells in the liver, simultaneously uptake and release a variety of molecules, such as glucose, proteins, and lipids to maintain appropriate blood chemistry; they also synthesize and secret bile for food digestion/absorption, cholesterol homeostasis, and toxin elimination (1). Like all epithelial cells, hepatocytes possess the apical (canalicular) and basolateral (sinusoidal) domains of the plasma membrane (PM) but are unique in that each hepatocyte is multipolarized with several apical domains in mature liver. The basolateral domain of a hepatocyte is shared with neighboring cells or faces fenestrated blood vessels called the sinusoids for substance exchange with blood, whereas the apical domain forms a common canaliculus for bile secretion (2). During fetal and neonatal liver development and in liver regenerating processes, hepatocytes appear to polarize and assemble as a single central lumen-containing cluster before becoming multipolarized for formation of anastomosed bile canaliculi (3, 4, 5, 6, 7).

In addition to the multipolarized phenotype, hepatocytes have also evolved a special membrane trafficking system to perform numerous vital functions (2, 8). In hepatocytes, but not in other epithelial cells, apical single-pass transmembrane proteins, such as dipeptidyl peptidase IV (DPP4), arrive from transcytosis: newly synthesized proteins are first recruited from the Golgi apparatus to the sinusoidal membrane before being targeted to the canalicular membrane. On the other hand, polytopic membrane proteins, including the ABC-cassette transporters ABCB1 and multidrug resistance protein 2 (MRP2), are directly delivered to the apical membrane of hepatocytes.

The border between the apical domain facing the lumen and the basolateral domain connecting to adjoining cells and extracellular matrix (ECM) is marked by the tight junction (TJ), which is linked to the machinery that regulates apicobasal polarization (9). The establishment of apicobasal polarity in epithelial cells requires signaling both from cadherin-mediated cell–cell interaction and from the ECM-elicited integrin activation (10, 11). The process involves an evolutionarily conserved network of polarity determinants, such as the Crb–PatJ–PALS1, Par3–Par6–aPKC, and Lgl–Dlg–Scribble complexes (9, 12). The transmembrane protein Crb3, complexed with the adaptor proteins PatJ and PALS1, probably provides apical membrane identity. The components are also linked to the Par complex, in which Par6 is tightly heterodimerized with the protein kinase aPKC *via* their PB1 domains (13); and the dimer directly binds to Par3, which contains three PDZ domains for protein–protein interaction (14). The Par3–Par6–aPKC complex and Cdc42 appear to promote TJ establishment and apical surface formation (15, 16). On the other hand, Lgl likely defines basolateral domain identity in cooperation with Dlg and Scribble (17, 18, 19, 20). Depletion of Par3, Par6, or aPKC in renal Madin–Darby canine kidney (MDCK) or intestinal Caco-2 epithelial cells leads to delayed TJ development in 2D culture (21, 22, 23, 24), whereas 3D culture of the depleted cells results in formation of aberrant cysts with multiple lumens, each surrounded by polarized cells, in contrast to a solitary apical lumen as observed in undepleted cells (25, 26, 27, 28). Similar phenotypes in both 2D and 3D cultures are observed in Cdc42-depleted MDCK cells (16). On the other hand, no lumen is formed in Lgl-depleted MDCK cells in 3D culture (19).

In contrast to a vast store of knowledge on the polarization of simple epithelial cells, it remains largely unknown how hepatocytes are polarized, except for the role of the cAMP-dependent pathway. Bile acid promotes hepatocyte polarization and bile canaliculus (BC) formation *via* elevation in an intracellular concentration of cAMP and subsequent activation of the protein kinases PKA and LKB1/Par4, the latter of which phosphorylates AMPK and its related kinases such as Par1b (29, 30, 31, 32). Although it has been reported that Par3 is present with ZO-1 at the outline of BC in rat liver tissue (33, 34) and participates in BC formation in rat hepatic Can 10 cells (35), the role of Par3 in hepatocyte polarity establishment has not been fully understood. Similarly, the hepatic functions of aPKC and Lgl have been hardly investigated. In addition, hepatocyte-specific deletion of Cdc42 is known to delay liver regeneration after partial hepatectomy in mice (36), but it has remained unclear whether this GTPase directly participates in hepatocyte polarization.

The present careful analysis reveals that, in addition to TJ enrichment of endogenous Par3, its binding protein aPKC localizes to both TJ and apical membrane in hepatocytes in mouse liver tissue and polarized human hepatocytic HepG2 cells. Using a 3D culture system with ECM-rich Matrigel, which enables HepG2 cells to efficiently polarize and form an apical (canalicular) lumen at the stage of two or a few cells, we show that Par3 plays a crucial role in hepatocyte polarity establishment. Indeed Par3-depleted HepG2 cells fail to initiate cell polarization and apical lumen formation, in contrast to the aforementioned phenotype of Par3-silenced MDCK cells, that is, multilumen cyst formation with cells, each retaining apicobasal polarity. Par3 and Cdc42 appear to recruit aPKC to the appropriate region of the PM for hepatocyte polarity establishment. The aPKC activity contributes not only to apical lumen formation but also to BC elongation in primary hepatocytes. Furthermore, the basolateral proteins Lgl1 and Lgl2 likely regulate lateral membrane integrity at least partially *via* restricting the localization of aPKC in hepatocytes.

## Results

### Expression and localization of cell polarity proteins in hepatocytes

**Figure 1:**
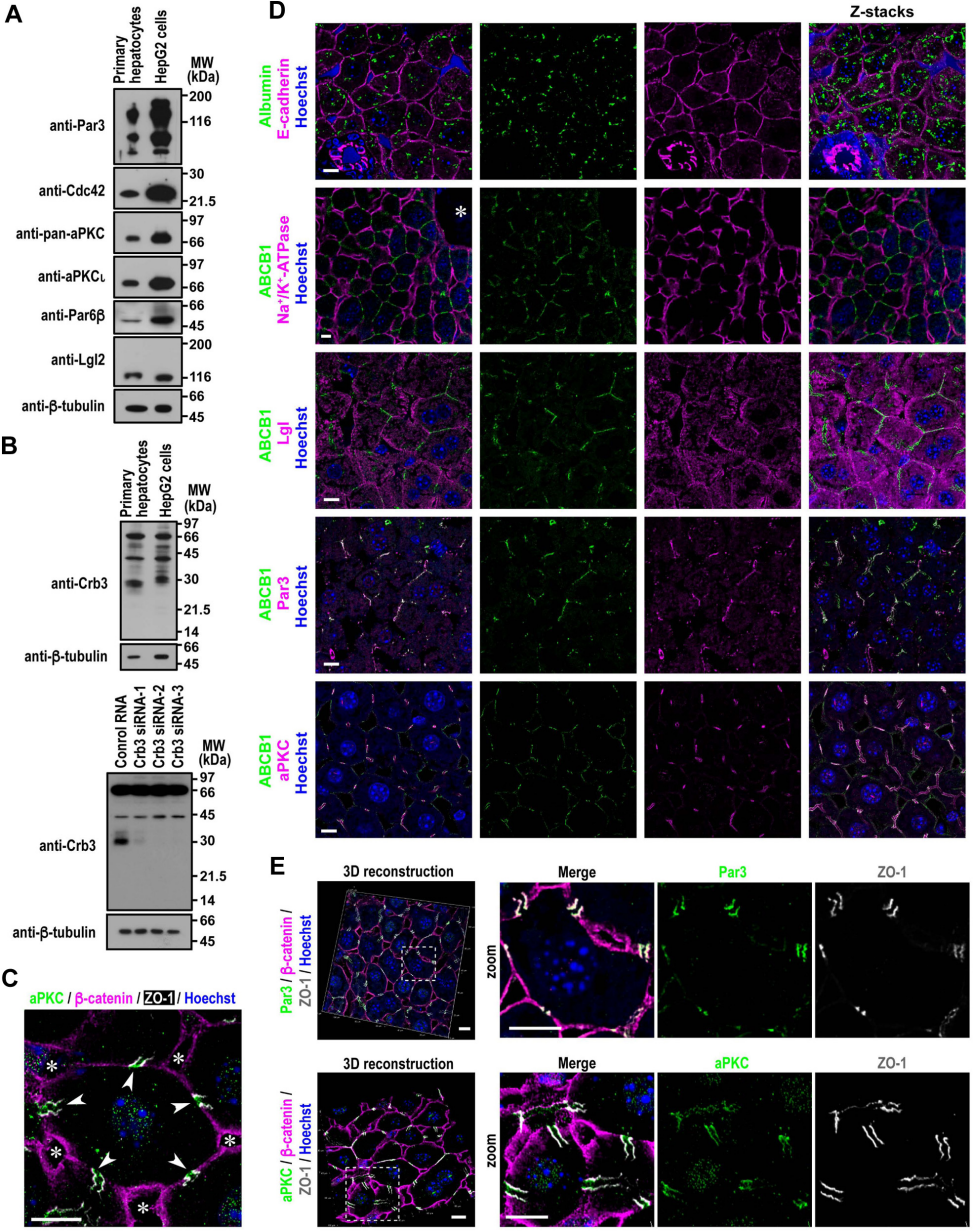
**Apicobasolateral polarity of hepatocytes in mouse liver tissue.***A*, proteins in lysates of mouse primary hepatocytes and HepG2 cells were analyzed by immunoblot with the indicated antibodies in three independent experiments. *B*, expression of Crb3. Proteins in the lysates of mouse primary hepatocytes and HepG2 cells transfected with control RNA or Crb3 siRNAs were analyzed by immunoblot with the anti-Crb3 antibody in three independent experiments. Positions for marker proteins are indicated in kilodalton. *C*, a representative image of a hepatocyte in mouse liver tissue. Paraffin-embedded sections of liver tissue isolated from 8-week-old male mice were stained with Hoechst and antibodies against aPKC, the basolateral protein β-catenin, and the TJ marker ZO-1. Confocal images were stacked along the *z*-axis. *Arrowheads* indicate BC; *asterisks* denote neighboring sinusoids. *D* and *E*, confocal (*D*) and 3D reconstruction (*E*) images of mouse liver tissue stained with the indicated antibodies and Hoechst. Stacked images along the *z*-axis (z-stacks) are also shown on the right-most panels in *D*. The *asterisk* indicates the central vein of the hepatic lobule. Magnifications of areas marked by the *dashed line* in (*E*) are presented in the respective *right panels* (zoom). The scale bars represent 10 μm. BC, bile canaliculus; TJ, tight junction.

To explore cell polarity proteins in hepatocytes, we first performed immunoblot analysis using lysates from mouse primary hepatocytes and human hepatocytic HepG2 cells (Fig. 1, *A* and *B*). The analysis revealed the expression of the following proteins: the TJ protein Par3, its interacting proteins Par6 and aPKC, the lateral identity protein Lgl, the apical membrane-integrated protein Crb3, and the small GTPase Cdc42, all of which are known to participate in polarization of nonhepatic epithelial cells at their respective regions (9, 37). Alternative splicing of the Par3 mRNA is known to yield three major proteins with a molecular mass of about 180, 150, and 100 kDa (38), and similar isoforms are expressed in the mouse liver and human hepatic cell lines including HepG2 (35, 39, 40), which agrees with the present analysis (Fig. 1*A*). Mouse Lgl2 (primary hepatocytes) migrated on SDS-PAGE a little more slowly than human Lgl2 (HepG2 cells) (Fig. 1*A*), as observed in a previous study (41). For detection of Crb3, we used a novel affinity-purified anti-Crb3 antibody (see Experimental procedures section), the authenticity of which was confirmed by a loss of the protein with a molecular mass of about 30 kDa, which corresponds with that of Crb3, in HepG2 cells transfected with Crb3-specific siRNAs (Fig. 1*B*).

We next investigated *in vivo* localization of the polarity proteins by confocal microscopy. In mouse liver tissue, the lateral domain of the PM of a hepatocyte is shared with neighboring cells and the basal domain faces fenestrated blood vessels called the sinusoids, whereas the apical domain exists at around the center of the lateral domain and forms a common BC. The canalicular or apical membrane domain in hepatocytes forms an about 2 μm band that encircles the cell surface and whose parallel margins are demarcated by TJs. Indeed ZO-1, a well-known TJ protein in hepatocytes (42), was clearly visualized as two parallel lines bordering the BC in the proximity of the center of cell–cell contact regions of the β-catenin-localized basolateral membrane (Fig. 1*C*). As shown in Figure 1*D*, hepatocytes, the sole albumin-producing cells, expressed the basolateral proteins E-cadherin and Na^+^/K^+^-ATPase on the almost entire PM but with several disconnected sites, which were occupied by the apical membrane protein ABCB1. Lgl was also present at the PM in a manner mutually exclusive with that of ABCB1, indicative of its basolateral localization in hepatocytes (Fig. 1*D*). The TJ protein ZO-1 is known to localize on the border of bile canaliculi in sections of mouse liver tissue (32, 34); similarly, the bile canaliculi stained with ABCB1 were bordered by Par3 (Fig. 1*D*). As shown by 3D reconstruction (Fig. 1*E*), Par3 colocalized with ZO-1, indicating the accumulation of Par3 at the TJ. The Par3-interacting protein aPKC was costained with ZO-1, as expected, but was also observed medial to the TJ, that is, the apical membrane that faces the BCs (Fig. 1, *C* and *E*). This finding is consistent with the colocalization of aPKC with the apical protein ABCB1 at least in part (Fig. 1*D*). Thus, aPKC appears to be present at both TJ and apical membrane in hepatocytes *in vivo*.

### Cell polarization and lumen formation in 3D-cultured HepG2 cells

**Figure 2:**
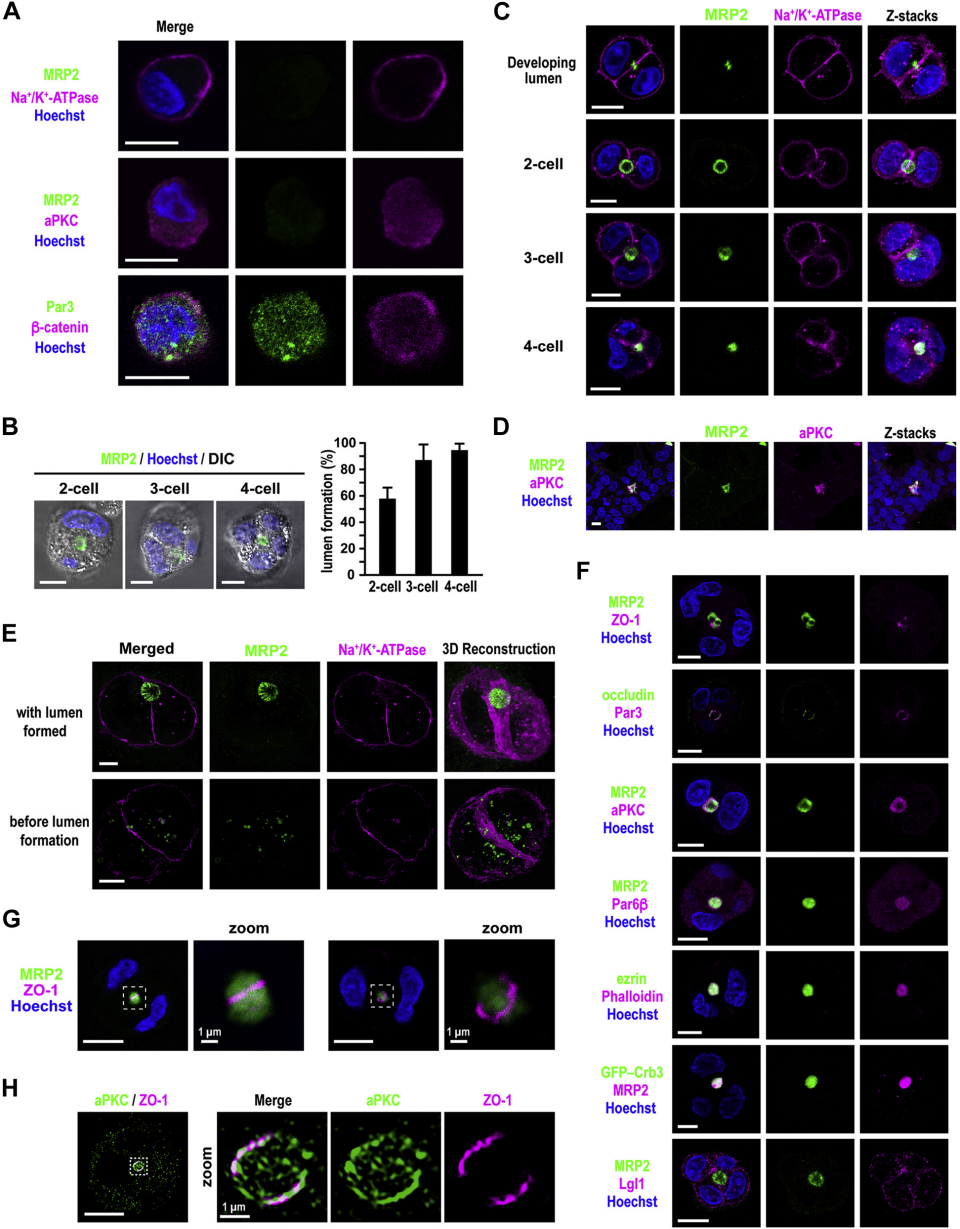
**Cell polarization and lumen formation in 3D-cultured HepG2 cells.***A*, representative confocal images of single HepG2 cells in suspension. Cells were fixed and stained with the indicated antibodies and Hoechst. *B*, representative differential interference contrast (DIC) images (*left*) and quantification of apical lumen formation (*right*) in 3D Matrigel culture of HepG2 cells. Cells were cultured for 24 h, followed by fixation and staining with the anti-MRP2 antibody and Hoechst. Cell clusters with a lumen were counted at the two-, three-, or four-cell stage (*n* ≥ 100 for each stage) in three independent experiments, and values are presented as the mean ± SD. *C* and *E–H*, representative confocal (*C*, *F*, and *G*) and super-resolution (*E* and *H*) images of 3D-cultured HepG2 cells. Cells were cultured for 24 h and stained with the indicated antibodies and Hoechst. The stacked images (z-stacks) are also shown on the right-most panels in *C*. The areas outlined with *dashed lines* in (*G*) and (*H*) are further magnified (zoom). *D*, HepG2 cells were cultured for 6 days and stained as indicated. The stacked images (z-stacks) are also shown. The scale bars represent 10 μm, unless otherwise indicated.

Human HepG2 cells, established from a differentiated hepatoma, provide a widely used model system for the study of cell polarization (2). To understand the mechanism for hepatocyte polarity establishment, we used HepG2 cells in 3D culture with ECM-rich Matrigel. For obtaining well-separated HepG2 cells for the culture, cells prepared from 2D monolayer culture were carefully passed through a 23-gauge needle (for details, see Experimental procedures section). The separated single cells in suspension appear to be unpolarized: Na^+^/K^+^-ATPase and β-catenin localized to the entire PM, and Par3 and aPKC distributed to the cytoplasm; and MRP2, a multimembrane-spanning protein that localizes apically in polarized HepG2 cells, was not detected (Fig. 2*A*).

In 3D Matrigel culture, HepG2 cells assembled to form central lumen–containing clusters, each comprising two to four cells, and MRP2 exclusively localized to the luminal membrane, indicative of cell polarization (Fig. 2, *B* and *C*). At the two-cell stage, lumen formation occurred in the center of the lateral membrane (Fig. 2, *B* and *C*). The central lumen was shared by multiple cells at later stages with three, four, and more cells (Fig. 2, *C* and *D*), which resembles an acinar structure observed during fetal and neonatal liver development and in liver regenerating processes after hepatectomy (3, 4, 5, 6). Further analysis by 3D structured illumination microscopy (3D-SIM), a super-resolution imaging technique, demonstrated the abundance in MRP2-positive and well-developed microvilli, indicating that the cells are fully polarized (Fig. 2*E*). On the other hand, no lumen was observed between a pair of yet-to-be polarized HepG2 cells, where MRP2 was distributed as vesicles in the cytoplasm of both cells (Fig. 2*E*).

The ZO-1- or occludin-stained TJs were observed as a linear or ring-like structure, depending on the confocal slice (Fig. 2, *F* and *G*). A similar observation was made with Par3 staining, and its colocalization with occludin confirmed Par3 as a TJ protein in polarized HepG2 cells (Fig. 2*F*). aPKC and its tightly associated partner Par6 were present not only at the apical membrane but also at the TJ, as indicated by the ring-like staining in addition to colocalization with the apical marker MRP2 (Fig. 2*F*). The conclusion is supported by super-resolution microscopic analysis demonstrating the colocalization of aPKC with ZO-1 (Fig. 2*H*). The luminal (apical) membrane was also rich in F-actin and its binding protein ezrin (Fig. 2*F*). Crb3, an apical membrane protein that participates in polarization of nonhepatic epithelial cells (37), was also presented on the apical domain when expressed as a GFP-fusion protein (Fig. 2*F*). On the other hand, Lgl localized to the entire nonapical membrane, indicating its identity as a basolateral protein (Fig. 2*F*). The polarity proteins thus localize to their own distinct cortical domains in 3D-cultured HepG2 cells as do they in hepatocytes *in vivo* (Fig. 1, *D* and *E*).

### Role of Par3 in hepatocyte polarization

**Figure 3:**
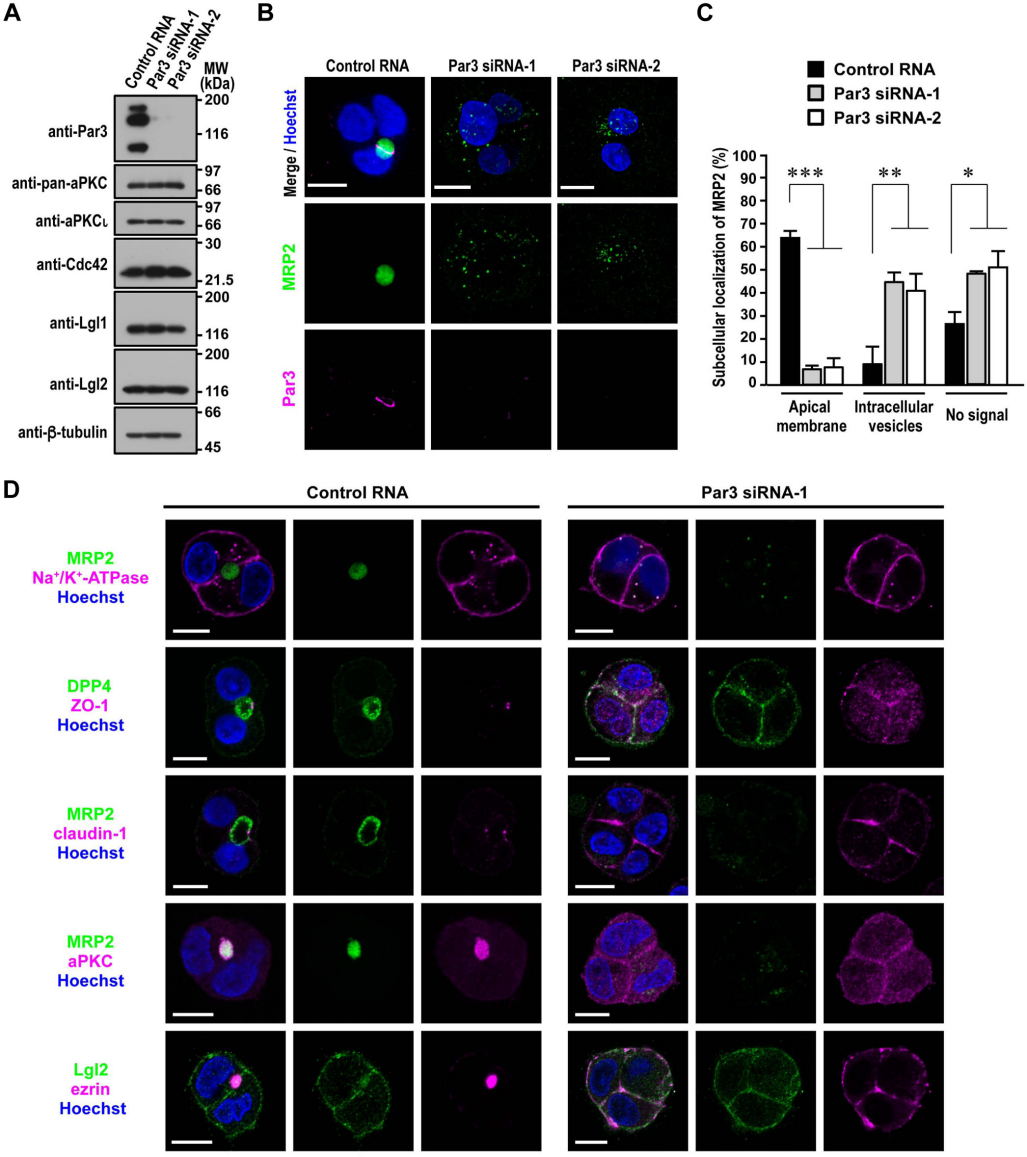
**Role of Par3 in hepatocyte polarization.***A*, depletion of Par3 in HepG2 cells. Proteins in lysates of HepG2 cells transfected with Par3-specific siRNA or control RNA were analyzed by immunoblot with the indicated antibodies in four independent experiments. Positions for marker proteins are indicated in kilodalton. *B* and *D*, representative confocal images of Par3-depleted HepG2 cells in 3D culture. Cells transfected with Par3-specific siRNA or control RNA were cultured for 24 h, followed by staining with the indicated antibodies and Hoechst. *C*, quantification of subcellular localization in Par3-depleted or control HepG2 cells in 3D culture. MRP2 localization was determined (*n* ≥ 100 for each condition) in three independent experiments, and values are presented as the mean ± SD. ∗*p* < 0.05; ∗∗*p* < 0.01; ∗∗∗*p* < 0.001 (one-way ANOVA followed by the Tukey–Kramer test). The scale bars represent 10 μm.

Although it is known that Par3 facilitates apicobasal polarization in kidney epithelial MDCK cells (25) and also participates in BC formation in rat hepatic Can 10 cells (35), further studies are required to know the role of Par3 in hepatocyte polarity establishment. For this purpose, we specifically depleted Par3 in HepG2 cells by RNA interference (Fig. 3*A*). Par3 depletion severely impaired apical lumen formation, as the signal for the apical membrane-integrated protein MRP2 was either almost lost or present at cytoplasmic vesicles but not at the PM (Fig. 3, *B* and *C*). DPP4 is a single-spanning membrane protein that is delivered initially to the lateral domain and subsequently to the apical domain by transcytosis in hepatocytes (8). Intriguingly, DPP4 localized exclusively to the apical membrane in most of control lumen–forming HepG2 cells (71%, *n* = 112) (Fig. 3*D*) but to the entire PM or the cytoplasm in Par3-depleted cells in a lumen-less cluster (81% or 19%, respectively, *n* = 116) (Fig. 3*D*). This is in contrast to complete exclusion from the PM of the polytopic transmembrane protein MRP2 (Fig. 3*B*), which is directly targeted to the apical domain in polarized hepatocytes (8). The knockdown of Par3 also led to cytoplasmic distribution of aPKC (100%, *n* = 115) and ezrin localization to the entire PM (88%, *n* = 105) (Fig. 3*D*). These observations indicate that Par3 plays a crucial role in hepatocyte polarity establishment and subsequent apical domain formation.

Furthermore, the lateral proteins Na^+^/K^+^-ATPase and Lgl became distributed to the entire PM in Par3-depleted cells, consistent with the absence of the apical domain (Fig. 3*D*). TJ development was also prevented: the membrane-integrated TJ component claudin was present throughout the entire PM. Importantly, the peripheral membrane protein ZO-1, which is normally targeted to the TJ and essential for TJ assembly (43), becomes distributed to the entire PM and cytoplasm (Fig. 3*D*). Although ZO-1 was concentrated at the TJ in 100% of lumen-forming control cells (*n* = 109), this protein distributed to the entire PM and cytoplasm in 83% of Par3-depleted cells that were uninvolved in lumen formation (*n* = 100), and no ZO-1 signal was detected in the rest of the cells (17% of Par3-depleted and lumen-uninvolved cells). Thus, Par3 depletion in HepG2 cells led to a failure of apicobasolateral polarity establishment. This phenotype is in sharp contrast to that of Par3-depleted MDCK cells, which retain the ability to polarize and thus form a cyst with multiple lumens, each surrounded by TJ-developed polarized cells in 3D culture (25, 26).

### Role of Cdc42 in hepatocyte polarization

Par3 interacts with Cdc42 *via* the Par6–aPKC dimer to regulate TJ development and subsequent formation of the apical membrane in 2D-cultured epithelial cells (44). In 3D culture of intestinal and renal epithelial cells, on the other hand, depletion of Cdc42 does not prevent the establishment of apicobasal polarity in individual cells but leads to multiple lumen formation (16, 45), which is also induced by knockdown of Par3, Par6, or aPKC (25, 27, 28).

**Figure 4:**
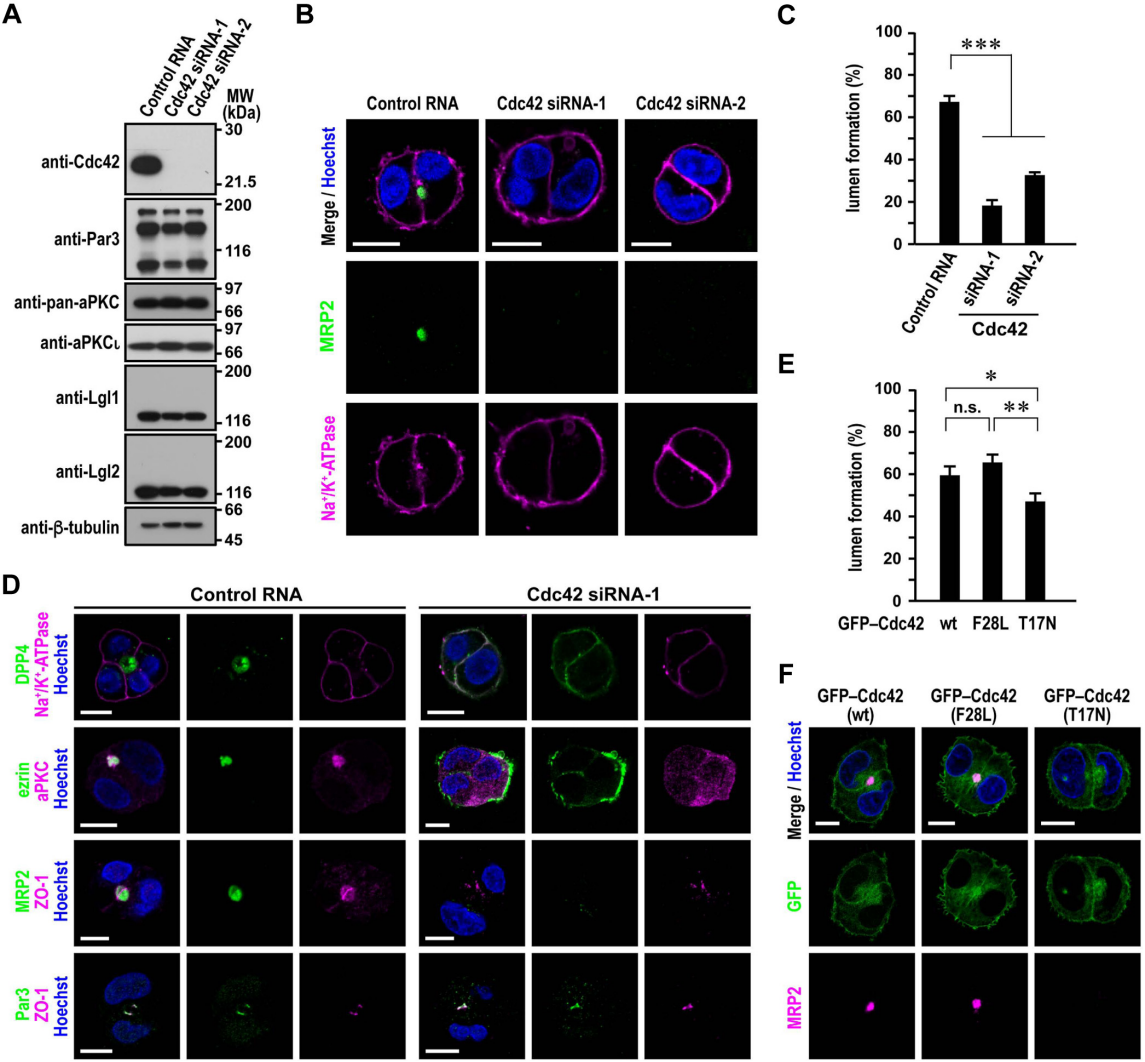
**Role of Cdc42 in hepatocyte polarization.***A*, depletion of Cdc42 in HepG2 cells. Proteins in lysates of HepG2 cells transfected with Cdc42-specific siRNA or control RNA were analyzed by immunoblot with the indicated antibodies in four independent experiments. Positions for marker proteins are indicated in kilodalton. *B* and *D*, representative confocal images of Cdc42-depleted HepG2 cells in 3D culture. Cells transfected with Cdc42-specific siRNA or control RNA were cultured for 24 h, followed by staining with the indicated antibodies and Hoechst. *C*, quantification of apical lumen formation in 3D culture of Cdc42-depleted or control HepG2 cells. Cell clusters (*n* ≥ 100 for each condition) were scored for lumen formation in three independent experiments, and values are presented as the mean ± SD. ∗∗∗*p* < 0.001 (one-way ANOVA followed by the Tukey–Kramer test). *E*, quantification of apical lumen formation in 3D culture of GFP–Cdc42-expressing HepG2 cells. Cell clusters (*n* ≥ 100 for each condition) were scored for lumen formation in three independent experiments, and values are presented as the mean ± SD. ∗*p* < 0.05; ∗∗*p* < 0.01 (one-way ANOVA followed by the Tukey–Kramer test). *F*, representative confocal images of HepG2 cells expressing GFP-tagged Cdc42 in 3D culture. Cells expressing GFP–Cdc42 (wt, F28L, or T17N) were cultured for 24 h, followed by staining with the indicated antibodies and Hoechst. The scale bars represent 10 μm. ns, not significant.

In HepG2 cells specifically depleted of Cdc42 (Fig. 4*A*), apical lumen formation was strongly impaired (Fig. 4, *B* and *C*): the multispanning membrane protein MRP2 was lost from the PM (Fig. 4*B*). In contrast, the bitopic transmembrane protein DPP4 and the peripheral membrane protein ezrin were distributed to the entire PM in 84% (*n* = 137) and 100% (*n* = 107) of Cdc42-depleted cells in a lumen-free cluster, respectively (Fig. 4*D*). The depletion resulted in cytoplasmic localization of aPKC (100%, *n* = 105) (Fig. 4*D*). On the other hand, both the TJ proteins Par3 and ZO-1 remained accumulated to cell–cell contact sites in 75% (*n* = 102) and 82% (*n* = 100) of Cdc42-depleted cells that do not participate in lumen formation, respectively (Fig. 4*D*). Cdc42 is thus crucial for hepatocyte polarity establishment but not required for recruitment of Par3 and ZO-1.

We further studied the role of Cdc42 by expressing various forms of Cdc42 as GFP-fused protein in HepG2 cells: wt, a GDP-fixed inactive form (T17N), and an active form with a facilitated GTP/GDP exchange rate (F28L) (46). Expression of Cdc42 (T17N) attenuated apical lumen formation (Fig. 4*E*), confirming the significance of the Cdc42 activity. Although Cdc42 (wt) and Cdc42 (T17N) distributed to the entire PM as well as the cytoplasm, Cdc42 (F28L) localized to the PM with accumulation at the apical domain (Fig. 4*F*), implying that GTP-bound Cdc42 acts at the apical membrane. It is thus likely that Cdc42 plays a crucial role in hepatocyte polarity establishment and apical lumen formation; in contrast, this GTPase is not involved in apicobasolateral polarization *per se* in intestinal and renal epithelial cells (16, 45, 47).

### Role of aPKC in hepatocyte polarization

**Figure 5:**
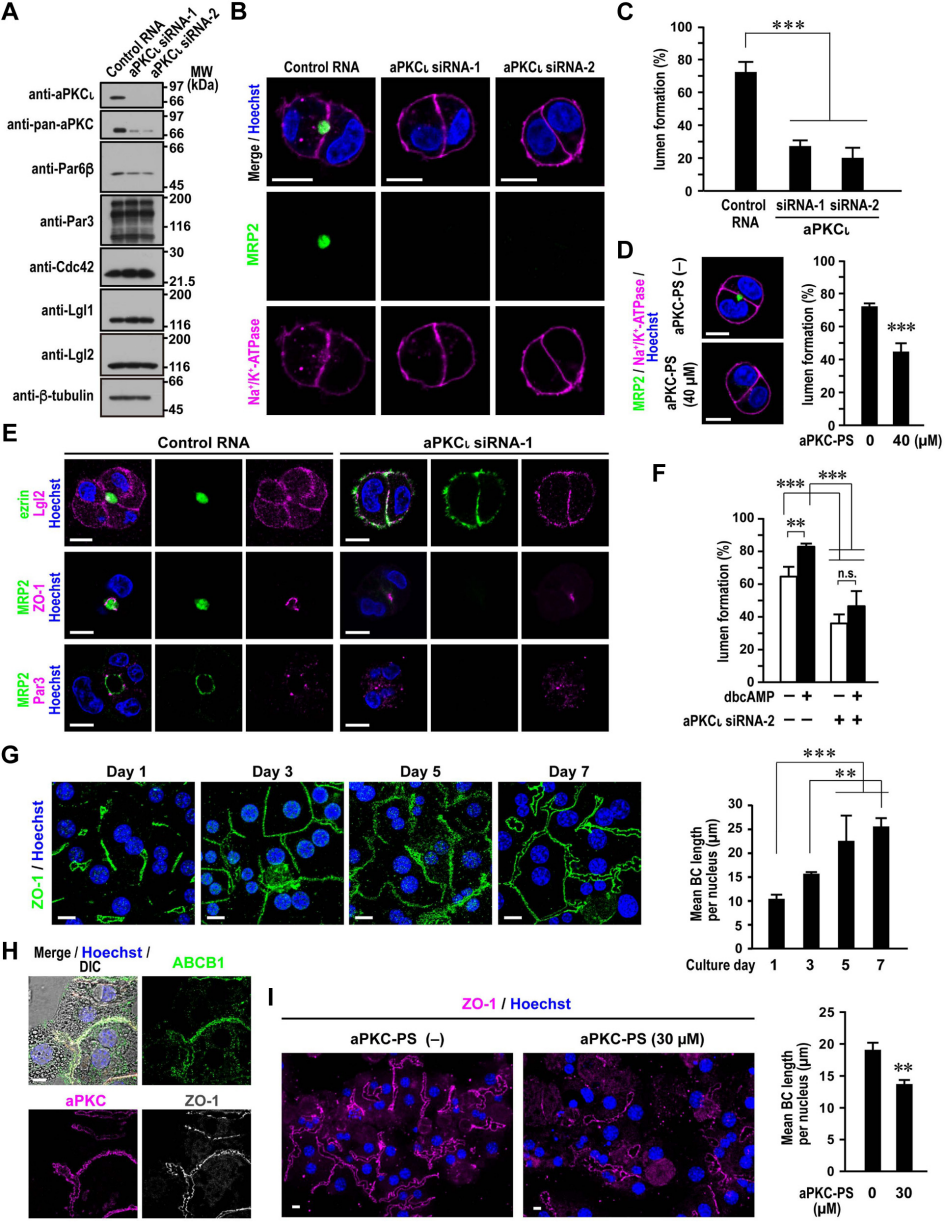
**Role of aPKC in hepatocyte polarization.***A*, depletion of aPKCι in HepG2 cells. Proteins in lysates of HepG2 cells transfected with aPKCι-specific siRNA or control RNA were analyzed by immunoblot with the indicated antibodies in four independent experiments. Positions for marker proteins are indicated in kilodalton. *B* and *E*, representative confocal images of aPKCι-depleted or control HepG2 cells in 3D culture. Cells were cultured for 24 h and stained with the indicated antibodies and Hoechst. *C*, quantification of apical lumen formation in 3D culture of aPKCι-depleted or control HepG2 cells. Cell clusters (*n* ≥ 100 for each condition) were scored for lumen formation in three independent experiments, and values are presented as the mean ± SD. ∗∗∗*p* < 0.001 (one-way ANOVA followed by the Tukey–Kramer test). *D*, representative confocal images (*left*) and quantification of apical lumen formation (*right*) in 3D culture of HepG2 cells treated with aPKC-PS. Cells were cultured for 24 h in the presence or absence of 40 μM aPKC-PS, followed by staining as indicated. Cell clusters (*n* ≥ 100 for each condition) were scored for lumen formation in three independent experiments, and values are presented as the mean ± SD. ∗∗∗*p* < 0.001 (Student's *t* test). *F*, quantification of apical lumen formation in 3D culture of HepG2 cells treated with dibutyryl-cAMP (dbcAMP). aPKCι-depleted or control cells were cultured for 24 h in the presence or absence of 1 mM dbcAMP. Cell clusters (*n* ≥ 100 for each condition) were scored for lumen formation in three independent experiments, and values are presented as the mean ± SD. ∗∗*p* < 0.01; ∗∗∗*p* < 0.001; ns, (one-way ANOVA followed by the Tukey–Kramer test). *G*, representative confocal images (*left*) and quantification of BC formation (*right*) in collagen sandwich culture of mouse primary hepatocytes. Hepatocytes isolated from 8-week-old male mice were cultured in a collagen gel sandwich for the indicated days, followed by staining as indicated. The total BC length in one image was divided by the total number of nuclei to calculate the mean BC length per nucleus (*n* ≥ 100 for each culture period). Values are mean ± SD of three independent experiments. ∗∗*p* < 0.01; ∗∗∗*p* < 0.001 (one-way ANOVA followed by the Tukey–Kramer test). *H*, representative differential interference contrast (DIC) and confocal images of primary hepatocytes. Cells were fixed at day 3 in collagen sandwich culture and stained as indicated. *I*, representative confocal images (*left*) and quantification of BC formation (*right*) in collagen sandwich culture of mouse primary hepatocytes treated with aPKC-PS. Hepatocytes were cultured in a collagen gel sandwich for 3 days in the presence or absence of 30 μM aPKC-PS, followed by fixation and staining as indicated. The total BC length in one image was divided by the total number of nuclei to calculate the mean BC length per nucleus (*n* ≥ 100 for each condition) in three independent experiments, and values are presented as the mean ± SD. ∗∗*p* < 0.01 (Student's *t* test). The scale bars represent 10 μm. BC, bile canaliculus; ns, not significant.

Because Cdc42 functions in apical membrane formation *via* its effector complex Par6–aPKC in kidney epithelial cells (44), we next studied the role of aPKC in hepatocyte polarization. Transfection of HepG2 cells with aPKCι-specific siRNAs led to a complete loss of this aPKC as shown by immunoblot analysis using its specific antibody (Fig. 5*A*). These cells lost a large part of aPKC detected by the pan-aPKC antibody that interacts with the two members of the mammalian aPKC family (aPKCι and aPKCζ), indicating that HepG2 cells mainly express aPKCι. Apical lumen formation was prevented by depletion of aPKCι (Fig. 5, *B* and *C*) and by cell treatment with aPKC-PS, an aPKC inhibitor (Fig. 5*D*). Thus, aPKC likely plays a crucial role in hepatocyte polarity establishment. The apical protein ezrin distributed to the entire PM in aPKCι-depleted HepG2 cells in a lumen-less cluster (100%, *n* = 101). On the other hand, the TJ proteins Par3 (89%, *n* = 100) and ZO-1 (82%, *n* = 101) remained concentrated in the center of the lateral membrane (Fig. 5*E*), suggesting that aPKC is not responsible for recruitment of Par3.

It is known that cAMP facilitates BC elongation, suggesting that cAMP is able to regulate hepatocyte polarization (48, 49). Indeed treatment of HepG2 cells with the cell-permeable analog dibutyryl-cAMP enhanced apical lumen formation (Fig. 5*F*). The formation was impaired by aPKC depletion, further supporting the importance of aPKC.

We also investigated the role of aPKC in primary hepatocytes. In collagen-sandwich culture of mouse primary hepatocytes, short BCs lining TJs were observed at day 1 and continued to lengthen up to day 7 (Fig. 5*G*). aPKC stained the canalicular membrane, colocalizing with both ZO-1 and the apical protein ABCB1 (Fig. 5*H*). Treatment with the aPKC inhibitor aPKC-PS reduced the length of BC (Fig. 5*I*), indicating the significance of aPKC in apical domain expansion in primary hepatocytes.

### Role of Lgl in hepatocyte polarization

**Figure 6:**
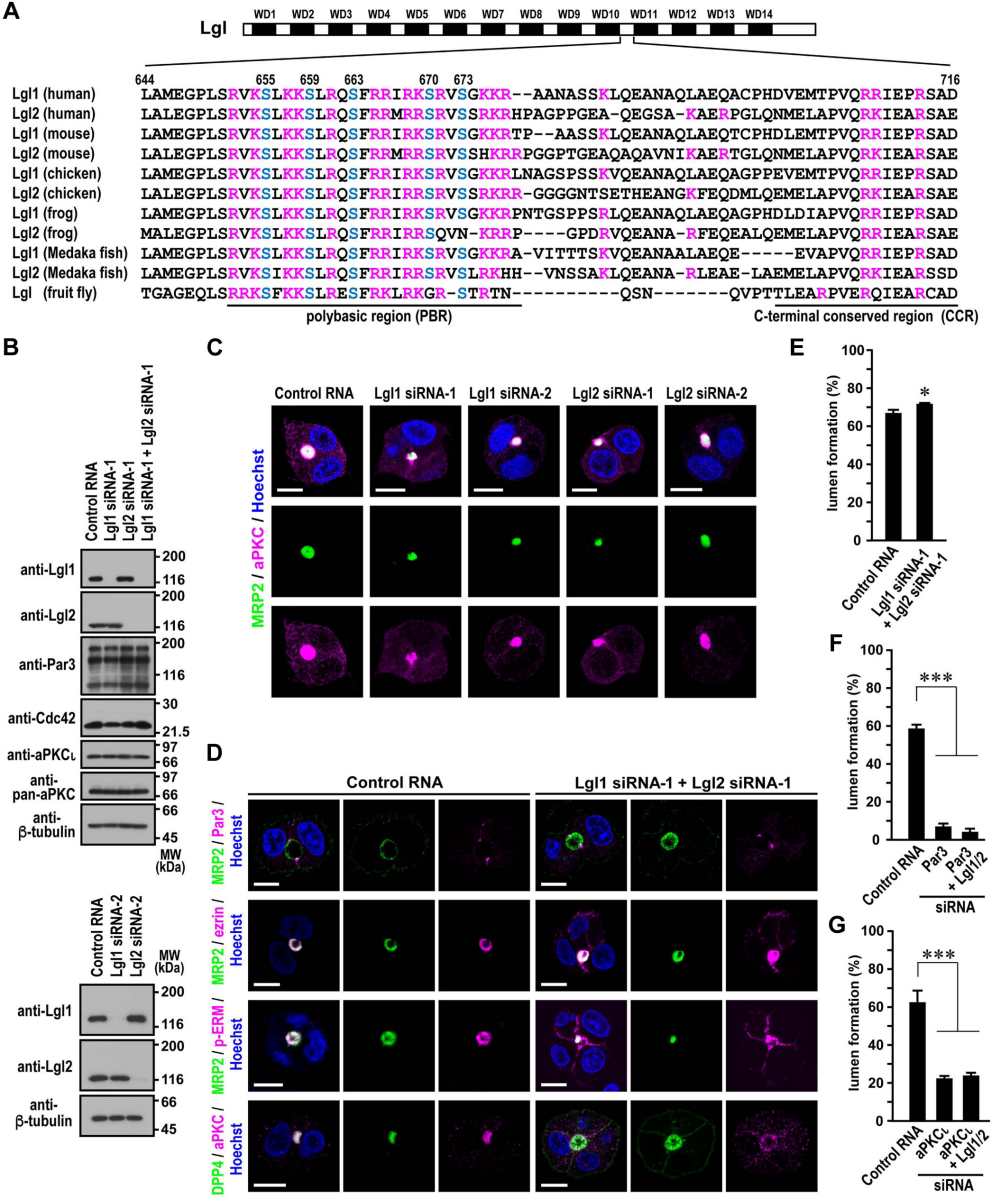
**Role for Lgl in regulation of lateral membrane integrity.***A*, domain organization of human Lgl1 and Lgl2 and sequence alignment of the PBR-containing loop in Lgl proteins from various species. The amino acid sequences of the loop region between the 10th and 11th WD40 blades are aligned: human, *Homo sapiens*; mouse, *Mus musculus*; chicken, *Gallus gallus*; frog, *Xenopus tropicalis*; Medaka fish *Oryzias latipes*; and fruit fly, *Drosophila melanogaster*. The basic residues lysine and arginine are shown in *magenta*, and the aPKC-phosphorylated serine residues are shown in *blue*. Numbers indicate amino acid positions in human Lgl1. *B*, depletion of Lgl1 and/or Lgl2 in HepG2 cells. Lysates of HepG2 cells transfected with the indicated siRNAs were analyzed by immunoblot with the indicated antibodies in four independent experiments. Positions for marker proteins are indicated in kilodalton. *C* and *D*, representative confocal images of Lgl-depleted HepG2 cells in 3D culture. Cells transfected with the indicated siRNAs were cultured for 24 h, followed by staining with the indicated antibodies and Hoechst. The scale bars represent 10 μm. *E–G*, quantification of apical lumen formation in 3D culture of Lgl-depleted HepG2 cells. After 3D cultured for 24 h, cell clusters (*n* ≥ 100 for each condition) were scored for lumen formation in three independent experiments, and values are presented as the mean ± SD. ∗*p* < 0.05 (Student's *t* test) (*E*); ∗∗∗*p* < 0.001 (one-way ANOVA followed by the Tukey–Kramer test) (*F* and *G*).

To identify aPKC substrates involved in hepatocyte polarization, we expressed the catalytically inactive aPKCι (K274E) (50) in HepG2 cells and analyzed its coprecipitated proteins by LC–MS/MS. The analysis demonstrated that Lgl1 and Lgl2 effectively bound to aPKCι (K274E) with their mass scores largely exceeding those of other known partners such as Par6 and Par3, and thus they were expected to be major substrates of aPKC in these cells (see Experimental procedures section). It is well known that the Lgl proteins, adopting a double β-propeller structure composed of 14 WD40 motifs (51), are evolutionarily conserved substrates of aPKC; aPKC binds to a loop between the 10th and 11th WD40 blades in the second propeller (Fig. 6*A*) and phosphorylates Ser655, Ser659, Ser663, Ser670, and Ser673 in Lgl1 and their respective residues in Lgl2 (17, 18, 52). The serine residues are present in a conserved polybasic region (PBR) in the N-terminal region of the loop, which recruits Lgl to the PM (53), whereas another conserved segment in the loop, the C-terminal conserved region (CCR), lacks net charge (Fig. 6*A*).

Specific depletion of Lgl1 or Lgl2 in HepG2 cells (Fig. 6*B*) did not affect apical localization of aPKC (Fig. 6*C*). Even in doubly Lgl-depleted cells, Par3 remained correctly targeted to the TJ, and apical lumen formation was marginally affected (Fig. 6, *D* and *E*). The double knockdown did not restore cell polarization impaired in Par3-or aPKCι-depleted cells (Fig. 6, *F* and *G*). The findings indicate that Lgl is dispensable for hepatocyte polarity establishment. In the absence of both Lgl1 and Lgl2, the membrane-integrated protein MRP2 remained strictly localized to the apical membrane, whereas the soluble proteins aPKC and ezrin crossed over to the lateral domain (Fig. 6*D*). The finding raises a possibility that Lgl participates in hepatocyte polarization *via* preventing aberrant apical protein localization to maintain lateral membrane integrity.

### Mechanism for control of lateral domain integrity by Lgl

**Figure 7:**
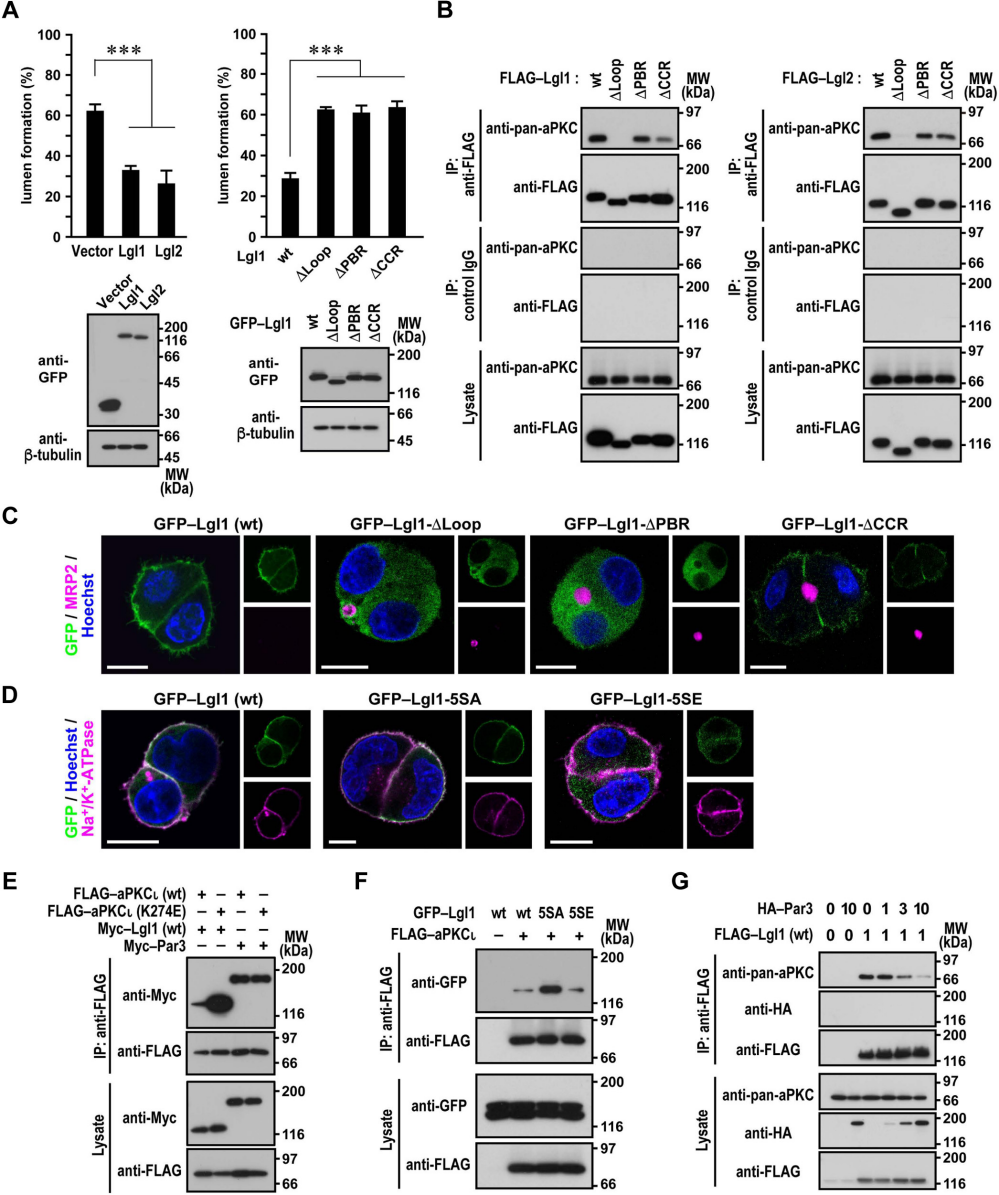
**Mechanism whereby Lgl regulates hepatocyte polarization.***A*, apical lumen formation and protein expression level of 3D-cultured HepG2 cells that overexpressed GFP-tagged Lgl proteins. Cells expressing the indicated Lgl protein were grown in 3D Matrigel culture for 24 h. Cell clusters (*n* ≥ 100 for each condition) were scored for lumen formation in three independent experiments, and values are presented as the mean ± SD (*upper panels*). ∗∗∗*p* < 0.001 (one-way ANOVA followed by the Tukey–Kramer test). Cell lysates were analyzed by immunoblot with the indicated antibodies in three independent experiments (*lower panels*). *B* and *E–G*, interaction of Lgl with aPKC. Proteins in the lysate of human embryonic kidney 293T (HEK293T) cells expressing the indicated proteins (lysate) were immunoprecipitated (IP) with the anti-FLAG M2 antibody or control IgG and then analyzed by immunoblot with the indicated antibodies in four independent experiments. The numbers for HA–Par3 and FLAG–Lgl1 in (*G*) indicate the relative amount of the plasmid DNA used for the transfection (0, 1, 3, or 10 times). Positions for marker proteins are indicated in kilodalton. *C* and *D*, representative confocal images of HepG2 cells expressing the indicated Lgl1 protein. Cells were grown in 3D culture for 24 h, followed by staining with the indicated antibodies and Hoechst. The scale bars represent 10 μm.

Consistent with the ability of Lgl to inhibit apical protein invasion (Fig. 6*D*), overexpression of Lgl1 or Lgl2 impaired apical lumen formation in HepG2 cells (Fig. 7*A*). A mutant Lgl1 lacking the loop region (Lgl1-Δloop), defective in binding to aPKC (Fig. 7*B*), failed to prevent apical lumen formation (Fig. 7*A*). Although Lgl1-ΔPBR and Lgl1-ΔCCR retained the ability to interact with aPKC (Fig. 7*B*), the apical lumen was fully formed in HepG2 cells overexpressing either mutant protein (Fig. 7*A*), indicating that both PBR and CCR in the loop region are required for Lgl-mediated control of lateral membrane integrity.

Prevention of aberrant apical protein localization by Lgl likely requires its localization to the lateral domain of the PM. It has been shown that, in nonhepatic cells, the PBR plays an essential role in Lgl recruitment to the PM *via* electrostatic binding to membrane phospholipids; the binding is abrogated by aPKC-catalyzed serine phosphorylation, which neutralizes the positive charges on the PBR to release Lgl from the PM (53, 54). In HepG2 cells, GFP–Lgl1 (wt) localized to both PM and cytoplasm, but truncation of the whole loop or the PBR alone resulted in exclusion from the PM (Fig. 7*C*), confirming the role of the PBR in hepatocytes. The phosphorylation-mediated regulation also appears to occur in HepG2 cells because GFP–Lgl1-5SA, in which the five PKC-phosphorylated serine residues (Ser655, Ser659, Ser663, Ser670, and Ser673) were replaced with the nonphosphorylatable residue alanine, was exclusively targeted to the PM, whereas Lgl1-5SE, carrying substitution of the phosphomimic residue glutamate for the serine residues, was observed in the cytoplasm (Fig. 7*D*). On the other hand, the requirement of the CCR for Lgl function (Fig. 7*A*) has not been thus far reported. In HepG2 cells, GFP–Lgl1-ΔCCR was enriched on the basolateral domain of the PM to an extent higher than that of Lgl1 (wt) (Fig. 7*C*), implying that the CCR attenuates the PBR-mediated association with the PM. Taken together, both positive and negative regulations of Lgl membrane recruitment by the PBR and CCR, respectively, are likely involved in control of lateral domain integrity in hepatocytes.

Localization of Lgl is also thought to be regulated by the TJ protein Par3 because Par3 is also a substrate of aPKC and thus competitively inhibits Lgl phosphorylation (14). As described in the present study, Lgl1 and Lgl2 but not Par3 are the major aPKCι (K274E)-binding proteins in HepG2 cells. FLAG–aPKCι (K274E) bound to Myc–Lgl1 to a much greater extent than that of Myc–Par3 (Fig. 7*E*), which agrees with the recent finding that aPKC phosphorylates Lgl more efficiently than Par3 *in vitro* (55). Myc–Lgl1 interacted strongly with catalytically inactive aPKCι (K274E) but only marginally with aPKC (wt) (Fig. 7*E*). As expected from the phosphorylation-induced effective dissociation of Lgl, aPKC binding to nonphosphorylatable Lgl1 (Lgl1-5SA) was stronger than that to Lgl1 (wt) (Fig. 7*F*). In contrast, Par3 bound to aPKCι (K274E) and to aPKCι (wt) to the same extent (Fig. 7*E*), indicating that phosphorylated Par3 does not easily dissociate from aPKC, probably because of its multiple interactions with the Par6–aPKC complex (44, 55, 56, 57). In agreement with the stable association with aPKC, Par3 efficiently released Lgl1 from aPKC (Fig. 7*G*). These findings suggest that Par3 antagonizes the action of Lgl at the TJ to regulate lateral domain integrity.

## Discussion

The polarity protein Par3 is enriched at the TJ, and its binding protein aPKC localizes to both TJ and apical membrane in hepatocytes in mouse liver tissue (Fig. 1) and polarized human hepatocytic HepG2 cells (Fig. 2). Using HepG2 cells in 3D Matrigel culture, we show that Par3 plays a crucial role in hepatocyte polarity establishment and subsequent apical lumen formation in conjunction with Cdc42 and aPKC (Figure 3, Figure 4, Figure 5). Par3 cooperates with ZO-1, an essential TJ-assembling protein (43), at a site for the apical lumen to be formed even in Cdc42- or aPKC-depleted HepG2 cells, whereas Par3 and Cdc42 are both required for PM localization of aPKC, which is responsible for apical development. The basolateral proteins Lgl1 and Lgl2 (Figs. 1 and 2), major aPKC substrates in hepatocytes, are dispensable for apical lumen formation but involved in maintenance of lateral domain integrity (Figs. 6 and 7).

The study on the molecular mechanism for hepatocyte polarization has been limited, compared with that for simple epithelial cells such as renal MDCK and intestinal Caco-2 cells, at least partly because of inefficient polarization of cultured hepatic cells. Here, we use HepG2 cells as an excellent model for the study of hepatocyte polarization (2): for microscopic analysis at the two or a few cell stages, carefully prepared single cells are used in 3D culture with Matrigel, which is known to provide a most suitable ECM for hepatocyte polarization at the single cell level (58) and in liver organoid formation (59). In HepG2 cells that become polarized under the present conditions (Fig. 2), all the marker proteins tested are correctly localized to the same membrane domains as those in mouse hepatocytes in liver tissue (Fig. 1). The central lumen with well-developed microvilli is usually formed at the two-cell stage, and subsequently shared by multiple polarized cells at later stages (Fig. 2), which resembles an acinar structure observed during fetal and neonatal liver development (3, 7) and in liver regeneration (4, 5, 6). Polarized hepatocytes contain the two distinct trafficking pathways for apical transmembrane proteins: the multispanning membrane protein MRP2 is directly targeted to the apical domain; and DPP4, a single-spanning membrane protein, is delivered initially to the lateral domain and subsequently transcytosed to the apical domain (2, 8). The two pathways are likely maintained in the present 3D-cultured HepG2 cells. MRP2 is not delivered to the PM in Par3-depleted HepG2 cells, which lack the apical membrane domain, the primary target site for MRP2; on the other hand, DPP4 is distributed to the entire PM because it is normally recruited to its primary target site, but not further transported in the absence of the apical membrane domain, its target site for transcytosis (Fig. 3). In MDCK cells, on the other hand, ectopically expressed DPP4 is directly transported to the apical domain but not by transcytosis (60, 61). The indirect targeting of apical proteins such as DPP4 is thus considered to be a hallmark of the hepatocytic polarity phenotype.

The present finding that depletion of Par3, Cdc42, or aPKC results in a loss of cell polarity and subsequent apical lumen formation in 3D-cultured HepG2 cells (Figure 3, Figure 4, Figure 5) is apparently different from that observed in 3D culture of renal MDCK or intestinal Caco-2 cells. In these epithelial cells, knockdown of either polarity protein results in formation of cysts with multiple lumens surrounded by polarized cells (16, 25, 27, 28, 45), indicating that cell polarization *per se* is not disturbed. The apparent difference is possibly because of the distinct unpolarized states at the one-cell stage. In a single unpolarized MDCK cell, the apical membrane-integrated protein gp135 as well as the basolateral protein β1-integrin is distributed all over the PM (62), whereas the otherwise apically localized protein MRP2 is absent from the PM in an unpolarized HepG2 cell (Fig. 2). Even at the two-cell stage, MRP2 distributes in the cytoplasm in yet-to-be unpolarized HepG2 cells (Fig. 2); in contrast, gp135 is initially present at the ECM-contacting surface in MDCK cells (47). It is thus tempting to postulate that the PM of hepatocytes but not that of other epithelial cells lacks the apical domain components as the default setting in an unpolarized state, and thus the polarity proteins Par3, Cdc42, and aPKC must exert their functions to a much greater extent for cell polarization and apical membrane development in hepatocytes. Basolateral membrane dominance in hepatocytes may be supported by the finding that depletion of the lateral identity protein Lgl in 3D culture does not perturb apical lumen formation in HepG2 cells (Fig. 7) but leads to defective lumen formation in other epithelial cells (19, 63). Consistent with this, Lgl overexpression in HepG2 cells, which is expected to further facilitate laterality dominance, prevents cell polarization and subsequent lumen formation (Fig. 7); on the other hand, in MDCK cells, Lgl overexpression induces multilumen cyst formation but does not affect apicobasal polarity of individual cells (25).

aPKC localizes to both TJ and apical (canalicular) membrane in polarized hepatocytes (Figs. 1 and 2). At the TJ, aPKC forms a stable complex with Par3, a protein that is required for aPKC localization (Fig. 3). On the apical membrane, instead, aPKC probably associates with GTP-bound Cdc42 since aPKC is excluded from the PM in Cdc42-depleted HepG2 cells (Fig. 4). In renal epithelial MDCK cells, the Par6–aPKC dimer localizes to the apical membrane *via* interaction with GTP-bound active Cdc42, and the apical membrane protein Crb3 (16). Crb3 is also expressed in hepatocytes and HepG2 cells, and GFP–Crb3 is efficiently targeted to the apical membrane in HepG2 cells (Figs. 1 and 2), and an active form of Cdc42 (F28L) is enriched at the apical membrane in HepG2 cells (Fig. 4). It seems thus possible that aPKC is recruited to the TJ *via* interacting with Par3 and to the apical membrane *via* assembling with Cdc42 and Crb3 in hepatocytes. During polarization of HepG2 cells, Par3 recruits the Par6–aPKC heterodimer to a site of the PM, which is then differentiated into the apical domain (Figs. 3 and 5); in response to apical activation by Cdc42, aPKC may dissociate from Par3, which thus demarcates the apical–lateral border, to drive differentiation and expansion of the apical membrane, as considered for the mechanism for polarization of simple epithelial cells (64). Interestingly, in *Caenorhabditis elegans* zygotes with anterior–posterior polarity, the localization of aPKC involves distinct and specialized aPKC-containing assemblies: a PAR-3-dependent assembly that responds to polarity cues and promotes efficient segregation of aPKC toward the anterior; and a CDC42-dependent assembly where aPKC is active but poorly segregated (65).

Inhibition of the aPKC activity as well as depletion of this enzyme impairs apical lumen formation in HepG2 cells (Fig. 5). The kinase activity is also involved in BC elongation in mouse primary hepatocytes (Fig. 5). The present analysis has identified the lateral proteins Lgl1 and Lgl2 as major substrates of aPKC in HepG2 cells (Fig. 7), and phosphorylation of Lgl likely drives its dissociation from hepatocyte PM (Fig. 7). Lgl-deficient MDCK cells in 3D culture fail to form an apical lumen (19). By contrast, in hepatocytes, Lgl1 and Lgl2 are dispensable for apical lumen formation (Fig. 6). Instead, the Lgl proteins appear to maintain lateral membrane integrity. In 3D-cultured HepG2 cells, depleted of both Lgl proteins, a considerable part of the soluble apical proteins aPKC and ezrin cross over to the lateral domain (Fig. 7), which is similar to the lateral invasion of the Par6–aPKC dimer observed in 2D culture of Lgl1/2-deficient colon epithelial cells (20). Consistent with the ability of Lgl to inhibit the invasion of apical proteins, overexpression of Lgl1 or Lgl2 prevents apical lumen formation in HepG2 cells (Fig. 7). Thus, Lgl likely functions *via* excluding the apical organizer aPKC from the lateral membrane in hepatocytes. Lateral integrity control in hepatocytes requires PM recruitment of Lgl, which is attenuated by the aPKC-catalyzed phosphorylation and is positively and negatively regulated by the PBR and the CCR, respectively (Fig. 7).

Par3 is also a substrate of aPKC (66). Intriguingly, after phosphorylation, Par3 remains associated with aPKC in contrast to Lgl (Fig. 7). The stable association is probably because of multiple interactions between Par3 and the Par6–aPKC heterodimer: Par3 directly binds to Par6 *via* the first and third PDZ domains (44, 55, 56) and to the aPKC C terminus *via* the second PDZ domain (57); Par3 also interacts with the aPKC catalytic domain *via* a C-terminal region to the third PDZ domain, an interaction that is released after phosphorylation (66, 67). In this stable complex, Par3 may regulate the aPKC activity by binding to the catalytic domain in competition with other substrates. This model explains well how aPKC stably localizes with Par3 at the TJ but displaces other substrates such as Lgl from the TJ and apical membrane.

It remains presently unclear how Par3 is selectively recruited and concentrated at an appropriate site of the PM to initiate cell polarization in hepatocytes as well as simple epithelial cells. Par3 recruitment can occur in a mitosis-dependent and mitosis-independent manner. The midbody formed during the first cell division appears to mark a site of nascent apical formation and function as an apical polarity cue in 3D-cultured MDCK cells (68); the apical membrane initiation site forms a ring structure around the midbody to act as a platform for delivery of apical proteins (69, 70). It has been reported that Par3 as well as ZO-1 localizes to the midbody just before abscission, the final stage of cell division, in rat hepatic Can 10 cells (35) and human endometrial epithelial cells (71). On the other hand, primary hepatocytes are capable of developing cell polarity without cell division: *de novo* polarization at the single-cell level is initiated by mere contact with both ECM and immobilized cadherin molecules defining a polarizing axis; this is followed by the accumulation of a dense actin cortex, Par3, and ZO-1 in the central region of the contact (58). Further investigations are required to clarify the molecular mechanism for membrane recruitment of Par3 for cell polarity establishment.

Hepatocytes differ from other epithelial cells in that each hepatocyte is multipolarized with several apical domains in mature liver (2). During development and regeneration of the liver, hepatocytes initially polarize and assemble as a single central lumen-containing cluster and are subsequently organized to become multipolarized for formation of anastomosed BC (3, 4, 5, 6, 7). Consistent with this, inhibition of aPKC results in blockade of both initial apical formation and BC elongation (Fig. 5). Depletion of Cdc42 also impairs hepatocyte polarization and apical lumen formation (Fig. 4), and Cdc42-deleted mice liver displays a disorganized lobular structure with dilated BCs in a regenerating process after hepatectomy (36). The detailed molecular mechanism for hepatocyte multipolarization should be investigated in future studies.

## Experimental procedures

### Antibodies and reagents

Rabbit antisera for Par6β were raised against the C-terminal 19-amino acid peptide of human Par6β (16). A mouse monoclonal antibody against ZO-1 (clone T8-754) was prepared as previously described (67, 72). Rabbit anti-Crb3a sera were raised against the C-terminal 20-amino acid peptide of human Crb3a, and the monospecific anti-Crb3a antibody was affinity purified using a HiTrap NHS-activated HP column (GE Healthcare Biosciences) conjugated with the immunogen. An anti-MRP2 mouse monoclonal antibody (clone M2 III-6; ab3373), an anti-Na^+^/K^+^-ATPase α1 subunit rabbit monoclonal antibody (clone EP1845Y; ab76020), and an anti-Lgl1 rabbit monoclonal antibody (clone EPR18899; ab183021), which cross-reacts with Lgl2 (20), were purchased from Abcam; anti-CD13 (clone 3D8; sc-13536), anti-MDR1/ABCB1 (clone D11; sc-55510), anti-Lgl2 (clone A-4; sc-376857), antialbumin (clone F-8; sc-374670) mouse monoclonal antibodies, an anti-ZO-1 rat monoclonal antibody (clone R40.76; sc-33725), anti-pan aPKC (anti-PKCζ; sc-216), and anti-β-catenin (sc-7199) rabbit polyclonal antibodies from Santa Cruz Biotechnology; anti-β-catenin (clone 14; 610154), anti-ezrin (clone 18; 610602), anti-Cdc42 (clone 44; 610929), and anti-PKCι/λ (clone 41; 610207) mouse monoclonal antibodies from BD Transduction Laboratory; anti-DPP4/CD26 (clone A6H; SAB4200230), anti-β-tubulin (clone TUB 2.1; T4026), and anti-FLAG (clone M2; F1804) mouse monoclonal antibodies and anti-FLAG rabbit polyclonal antibodies (F7425) from Sigma–Aldrich; anti-ezrin (07-130) and anti-Par3 (07-330) rabbit polyclonal antibodies from Millipore; anti-phospho-ezrin (Thr567)/radixin (Thr564)/moesin (Thr558) (P-ERM) (clone 48G2; 3726), anti-Lgl1 (clone D2B5A; 12159), and anti-E-cadherin (clone 24E10; 3195) rabbit monoclonal antibodies from Cell Signaling Technology; an anti-occludin (clone OC-3F10; 33-1500) mouse monoclonal antibody, and anti-ZO-1 (61-7300) and anti-claudin-1 (51-9000) rabbit polyclonal antibodies from Thermo Fisher Scientific; an anti-ABCB1/P-glycoprotein mouse monoclonal antibody (clone C219; ALX-801-002) from Enzo; an anti-HA mouse monoclonal antibody (clone 16B12; MMS-101P) from Covance; an anti-Myc mouse monoclonal antibody (clone 9E10) from Roche Applied Science; and an anti-GFP mouse monoclonal antibody (GF200; 04363-24) from Nacalai Tesque. Myristoylated PKC-Zeta Pseudosubstrate (77749), an inhibitor for aPKC, was obtained from Thermo Fisher Scientific.

### Plasmids

The complementary DNA (cDNA) for human Par3 was obtained by PCR using a human fetal brain cDNA library (Stratagene) (67). The cDNAs for human aPKCι and Cdc42 were obtained by RT–PCR using RNAs prepared from SH-SY5Y cells and the human leukemia K562 cells, respectively (50, 73). The cDNAs encoding human Lgl1 and Lgl2 were obtained by PCR using a human fetal brain cDNA library (Takara Bio, Inc) and an EST clone (cDNA clone MGC 75113 IMAGE: 5575350), respectively. Mutations leading to the indicated amino acid substitutions or deletions were introduced by PCR-mediated site-directed mutagenesis. The cDNA encoding full-length human Crb3a (amino acid residues 1–120) was obtained by PCR using kidney cDNAs (Human Multiple Tissue cDNA Panel; Takara Bio, Inc) as previously described (16). For expression of GFP–Crb3, the cDNA encoding enhanced GFP was inserted between the cDNA sequence for the signal peptide and that for the mature protein of human Crb3a. The cDNAs were ligated to the following expression vectors: pEF-BOS (74) or pcDNA3.1(+) (Thermo Fisher Scientific) for expression in mammalian cells; and pEGFP-C1 (Takara Bio, Inc) for expression as an N-terminally GFP-tagged protein in mammalian cells. All the constructs were sequenced for confirmation of their identities.

### Preparation of mouse liver tissue and primary hepatocytes

The present mouse studies were all conducted with the approval of Kyushu University (identification code: A20-090-0) and were performed in accordance with the guidelines of the Committee of Ethics on Animal Experiments, Faculty of Medical Sciences, Kyushu University. We obtained 8-week-old male mice (Jcl:ICR) from CLEA Japan, Inc. Preparation of the liver tissue and primary hepatocytes was performed according to the method of Sekiya and Suzuki (75) with minor modifications. In brief, after anesthesia with sevoflurane inhalation combined with an intraperitoneal injection of pentobarbital (40 mg/kg), the liver was perfused through the portal vein first with buffer A (137 mM NaCl, 5 mM KCl, 0.5 mM NaH_2_PO_4_, 0.8 mM Na_2_HPO_4_, 0.5 mM EGTA, 4 mM NaHCO_3_, 5 mM d-glucose, and 10 mM Hepes, pH 7.2), then treated with 0.5 mg/ml of collagenase type IV (Worthington) in Hank's solution (Nissui) supplemented with 4 mM CaCl_2_, 4 mM NaHCO_3_, and 10 mM Hepes, pH 7.5. The collagenase-treated liver was resected, and cells were released by tearing the Glisson's capsule. After centrifugation three times for 1 min at 50*g*, cells were resuspended in hepatomedium, a 1:1 mixture of Dulbecco's modified Eagle's medium and F-12 (Gibco), supplemented with 10% fetal bovine serum (FBS), 1 μg/ml of insulin (FUJIFILM Wako), 0.1 μM dexamethasone (Nacalai Tesque), 10 mM nicotinamide (FUJIFILM Wako), 50 μM β-mercaptoethanol (Nacalai Tesque), and penicillin/streptomycin. Cells were stained with trypan blue to assess cell viability and plated on the 14-mm diameter surface of a glass bottom dish (No.1S; Matsunami Glass) coated with collagen I (Nitta Gelatin) for experiments as indicated later. For tissue immunohistochemistry experiments, after perfusion with buffer A, the liver was fixed with a solution of 4% paraformaldehyde, resected, and kept overnight in the fixing solution.

### Cell culture and transfection

Primary hepatocytes were plated on glass bottom dishes coated with collagen I. After overnight incubation at 37 °C under 5% CO_2_, hepatocytes were overlaid with collagen I and further cultured in hepatomedium for the indicated days. HepG2 cells (American Type Culture Collection) were cultured in Eagle's minimum essential medium (MEM) (Nissui) supplemented with 10% FBS and 60 mg/l of kanamycin. Human embryonic kidney 293T (HEK293T) cells (American Type Culture Collection) were cultured in Dulbecco's modified Eagle's medium (Nissui) supplemented with 10% FBS and penicillin/streptomycin. Transfection of HepG2 or HEK293T cells with cDNAs was performed with Lipofectamine3000 reagent (Thermo Fisher Scientific) or X-tremeGENE HP transfection reagent (Roche), respectively. The transfected cells were cultured for 48 h before use in further analysis.

### 3D Matrigel culture of HepG2 cells

HepG2 cells were incubated in PBS (137 mM NaCl, 2.7 mM KCl, 8.1 mM Na_2_HPO_4_, and 1.5 mM KH_2_PO_4_, pH 7.4) containing 1 mM EDTA for 7 min at 37 °C, and then treated with 0.125% trypsin solution for 2 min. Cells were collected in MEM medium and passed through a 23-gauge needle 15 times to obtain single cells, after which cells were centrifuged, resuspended in fresh medium, and counted. About 8-well chambered cover glasses (Matsunami Glass or IWAKI) were coated with 40 μl/well of cold 100% Matrigel (Corning), rich in collagen IV and laminin, and allowed to gel for 5 to 10 min at 37 °C, and 300 μl of the cell suspension was plated at a density of 2.0 × 10^4^ cells/well. Cells were cultured for 24 h, followed by fixation. For dibutyryl-cAMP treatment, the plated cells were cultured for 24 h in the presence of 1 mM *N*^6^,2-*O*-dibutyryladenosine 3′,5′-cyclic monophosphate sodium salt (Tokyo Kasei) in MEM with 1% FBS, before fixation.

### Immunofluorescence microscopy

Cells cultured in Matrigel or a collagen sandwich were fixed with 4% paraformaldehyde for 30 min at room temperature, with 100% methanol for 10 min at −20 °C, or with 10% trichloroacetic acid for 3 min at room temperature, depending on the primary antibody used. Fixed cells were then treated with a blocking solution containing 3% bovine serum albumin (BSA) dissolved in PBS for 30 min. In the case of paraformaldehyde fixation, cells were further permeabilized with a solution containing 0.5% Triton X-100 and 3% BSA dissolved in PBS. The samples were incubated with primary antibodies for a period of 1 to 7 days, then washed, and incubated with the following secondary antibodies: Alexa Fluor 488-labeled goat anti-rabbit or anti-mouse IgG antibodies; Alexa Fluor 594-labeled anti-rabbit or anti-mouse IgG antibodies; and Alexa Fluor 633-labeled anti-mouse or anti-rat IgG antibodies (Thermo Fisher Scientific). Actin filaments were stained with Alexa Fluor 647 phalloidin (Thermo Fisher Scientific), and nuclei were stained with Hoechst 33342 (Thermo Fisher Scientific). For liver tissue immunohistochemistry, fixed liver tissue blocks were embedded in paraffin, sectioned in 5 to 15 μm slices with a microtome, and mounted on slides. Paraffin wax was removed with a series of xylene and ethanol, and then antigen retrieval was performed by boiling the slides in a buffer containing 50 mM Tris–HCl (pH 9.5). Samples were blocked for 30 min with a solution containing 3% BSA dissolved in PBS. After overnight incubation at 4 °C in a humidified chamber with the corresponding primary antibodies, samples were washed three times in PBS, then incubated with secondary antibodies for 1 h at room temperature, and nuclei were stained with Hoechst 33342 for 10 min. After washing with PBS, samples were mounted with coverslips using Vectashield HardSet Antifade mounting medium (Vector Laboratories). Confocal images were captured at room temperature with the confocal microscopes LSM700 (Carl Zeiss), LSM780 (Carl Zeiss), or A1R HD25 (Nikon), followed by analysis with ZEN (Carl Zeiss) or NIS elements (Nikon). The microscope LSM700 was equipped with a Plan-Apochromat 20×/0.8 numerical aperture (NA) dry objective lens and a Plan-Apochromat 63×/1.4 NA oil-immersion objective lens. The microscope LSM780 was equipped with a Plan-Apochromat 40×/1.3 NA oil-immersion and a C-Apochromat 63×/1.2 NA water-immersion objective lens. The microscope A1R HD25 was equipped with a Plan-Apochromat 40×/1.25 NA silicon-immersion objective lens and a Plan Apochromat 60×/1.27 NA water-immersion objective lens. For preparation of figures, images were edited with the GNU Image Manipulation Program (GIMP 2.10.10; www.gimp.org) and ImageJ 1.52 (National Institutes of Health) software.

### Super-resolution SIM imaging and analysis

SIM images were taken with an N-SIM system (Nikon) attached to a Ti2-E inverted microscope (Nikon) with a CMOS camera (ORCA-Flash 4.0 V3; Hamamatsu Photonics) using a Plan Apochromat 100×/1.35 NA silicon-immersion objective lens at a step size of 0.12 μm. The chromatic aberration of the system was calibrated and corrected by 0.1 μm TetraSpeck beads (T7279; Thermo Fisher Scientific) in the mounting medium. Images were reconstructed and analyzed using NIS elements AR (Nikon) according to the manufacturer's protocol.

### Analysis of apical lumen formation in 3D culture of HepG2 cells

After HepG2 cells were cultured in 3D Matrigel culture, cell clusters, each comprising two to four cells, were stained with the anti-MRP2 antibody and Hoechst to define the apical lumen and nuclei, respectively. HepG2 cell clusters were scored for apical lumen formation (*n* ≥ 100 for each condition) in three independent experiments.

### Measurement of BC length in primary hepatocytes

Confocal images of stained hepatocytes in the primary culture were obtained with LSM780 (Carl Zeiss) as described previously and stacked on the *z*-axis. Canalicular length was measured using ImageJ 1.52 software, then summed, and divided by the total number of nuclei to obtain the BC length per nucleus. Counts were performed in triplicate for each experimental condition.

### Protein identification by LC–tandem MS (MS/MS) analysis

HepG2 cells were transfected with a plasmid vector encoding FLAG-tagged aPKCι (K274E), a mutant lacking the kinase activity, or with an empty FLAG vector and cultured for 48 h. Cells were lysed at 4 °C with a lysis buffer (150 mM NaCl, 5 mM EDTA, 1 mM DTT, 0.01% Triton X-100, 10% glycerol, and 50 mM Tris–HCl, pH 7.5) containing Protease Inhibitor Cocktail (Sigma–Aldrich). Proteins in the lysates were precipitated with the anti-FLAG M2 antibody (M2)-conjugated magnetic beads (Sigma–Aldrich). After washing three times with the lysis buffer, the proteins were subjected to SDS-PAGE, followed by silver staining. Protein identification using LC–MS/MS was performed at the Laboratory for Research Support, Medical Institute of Bioregulation, Kyushu University, according to the protocol of Matsumoto *et al.* (76). Bands separated by SDS-PAGE were excised from the gel, and the proteins in the gel were digested with Trypsin gold Mass Spectrometry grade (PROMEGA) dissolved in 25 mM ammonium bicarbonate solution. The gel-extracted peptides were subjected to nano-LC–MS/MS analysis using an LTQ Orbitrap Velos Pro mass spectrometer system (Thermo Fisher Scientific) coupled with an Advance UHPLC system (Bruker) and an HTC-PAL autosampler (CTC Analytics). MS/MS spectra were obtained automatically in a data-dependent scan mode and compared with those in the UniProtKB/SwissProt human peptide database (UniProt Consortium) using the MASCOT search engine (Matrix Science). Assigned high-scoring peptide sequences were manually confirmed by comparison with the corresponding spectra.

### Knockdown with siRNA

Double-stranded siRNAs targeting human Crb3, Cdc42, Par3, aPKCι, Lgl1, and Lgl2 containing the following sequences on the sense strand of 25-nucleotide modified synthetic RNAs (Stealth RNAi; Thermo Fisher Scientific) were used: Crb3 siRNA-1, 5′-CCAUCACUGCUAUCAUCGUGGUCUU-3′ (sense) and 5′-AAGACCACGAUGAUAGCAGUGAUGG-3′ (antisense); Crb3 siRNA-2, 5′-CAGGGAAGAAGGUACUUCAAAGACU-3′ (sense) and 5′-AGUCUUUGAAGUACCUUCUUCCCUG-3′ (antisense); Crb3 siRNA-3, 5′-CAGUCAGAUCCACCCAGUGCUUAAU-3′ (sense) and 5′-AUUAAGCACUGGGUGGAUCUGACUG-3′ (antisense); Par3 siRNA-1, 5′-CCAUGUGGUUCCUGCAGCAAAUAAA-3′ (sense) and 5′-UUUAUUUGCUGCAGGAACCACAUGG-3′ (antisense); Par3 siRNA-2, 5′-GGCUUCGGGUGAAUGAUCAACUGAU-3′ (sense) and 5′-AUCAGUUGAUCAUUCACCCGAAGCC-3′ (antisense); Cdc42 siRNA-1, 5′-CCUCUACUAUUGAGAAACUUGCCAA-3′ (sense) and 5′-UUGGCAAGUUUCUCAAUAGUAGAGG-3′ (antisense); Cdc42 siRNA-2, 5′-UCCUUUCUUGCUUGUUGGGACUCAA-3′ (sense) and 5′-UUGAGUCCCAACAAGCAAGAAAGGA-3′ (antisense); aPKCι siRNA-1, 5′-ACUUCCUGAAGAACAUGCCAGAUUU-3′ (sense) and 5′-AAAUCUGGCAUGUUCUUCAGGAAGU-3′ (antisense); aPKCι siRNA-2, 5′-GAAGAUUGAUCAGUCUGAAUUUGAA-3′ (sense) and 5′-UUCAAAUUCAGACUGAUCAAUCUUC-3′ (antisense); Lgl1 siRNA-1, 5′-CAGCCACUGUCACACAGAUGCACUU-3′ (sense) and 5′-AAGUGCAUCUGUGUGACAGUGGCUG-3′ (antisense); Lgl1 siRNA-2, 5′-CAAGAUUCUGUGGCGGAACUGUGAA-3′ (sense) and 5′-UUCACAGUUCCGCCACAGAAUCUUG-3′ (antisense); Lgl2 siRNA-1, 5′-UGGAUGACAACAGCCUGCACCUUUG-3′ (sense) and 5′-CAAAGGUGCAGGCUGUUGUCAUCCA-3′ (antisense); and Lgl2 siRNA-2, 5′-GCGAUUACCAGAAUCCUCUGGCUGA-3′ (sense) and 5′-UCAGCCAGAGGAUUCUGGUAAUCGC-3′ (antisense). Low GC Duplex of Stealth RNAi Negative Control Duplexes #2 (Thermo Fisher Scientific) was used as a negative control. HepG2 cells plated at a density of 3.6 × 10^4^/cm^2^ were transfected with 20 nM siRNA using Lipofectamine RNAi MAX (Thermo Fisher Scientific) and cultured for 24 h before use in further analysis.

### Immunoprecipitation assay

Transfected HEK293T cells were lysed at 4 °C with lysis buffer 1 (150 mM NaCl, 5 mM EDTA, 1 mM DTT, 0.1% Triton X-100, 10% glycerol, and 50 mM Tris–HCl, pH 7.5) or lysis buffer 2 (150 mM NaCl, 5 mM EGTA, 2 mM MgCl_2_, 10 mM β-glycerophosphate, 5 mM NaF, 1 mM NaPPi, 1 mM Na_3_VO_5_, 1 mM DTT, 0.1% Triton X-100, 10% glycerol, and 50 mM Tris–HCl, pH 7.5) containing Protease Inhibitor Cocktail. The lysates were mixed with the indicated antibodies and precipitated with protein G-Sepharose (GE Healthcare). The precipitants were analyzed by immunoblot with the indicated antibodies, and the blots were developed using ImmunoStar Zeta or ImmunoStar LD (FUJIFILM Wako) for visualization.

### Statistical analysis

Statistical analyses were performed with R software, version 3.6.1 (The R Foundation) using the Student's *t* test or one-way ANOVA followed by the Tukey–Kramer test.

## Data availability

All the data are contained within the article.

## Conflict of interest

The authors declare that they have no conflicts of interest with the contents of this article.

## References

[1] Trefts E., Gannon M., Wasserman D.H. (2017). The liver. Curr. Biol..

[2] Treyer A., Müsch A. (2013). Hepatocyte polarity. Compr. Physiol..

[3] Wood R.L. (1965). An electron microscope study of developing bile canaliculi in the rat. Anat. Rec..

[4] Dezső K., Nagy P., Paku S. (2020). Human liver regeneration following massive hepatic necrosis: Two distinct patterns. J. Gastroenterol. Hepatol..

[5] Stamatoglou S.C., Enrich C., Manson M.M., Hughes R.C. (1992). Temporal changes in the expression and distribution of adhesion molecules during liver development and regeneration. J. Cell Biol..

[6] Ogawa K., Medline A., Farber E. (1979). Sequential analysis of hepatic carcinogenesis: The comparative architecture of preneoplastic, malignant, prenatal, postnatal and regenerating liver. Br. J. Cancer.

[7] Feracci H., Connolly T.P., Margolis R.N., Hubbard A.L. (1987). The establishment of hepatocyte cell surface polarity during fetal liver development. Dev. Biol..

[8] Schulze R.J., Schott M.B., Casey C.A., Tuma P.L., McNiven M.A. (2019). The cell biology of the hepatocyte: A membrane trafficking machine. J. Cell Biol..

[9] Rodriguez-Boulan E., Macara I.G. (2014). Organization and execution of the epithelial polarity programme. Nat. Rev. Mol. Cell Biol..

[10] Akhtar N., Streuli C.H. (2013). An integrin–ILK–microtubule network orients cell polarity and lumen formation in glandular epithelium. Nat. Cell Biol..

[11] Bryant D.M., Roignot J., Datta A., Overeem A.W., Kim M., Yu W., Peng X., Eastburn D.J., Ewald A.J., Werb Z., Mostov K.E. (2014). A molecular switch for the orientation of epithelial cell polarization. Dev. Cell.

[12] Wen W., Zhang M. (2018). Protein complex assemblies in epithelial cell polarity and asymmetric cell division. J. Mol. Biol..

[13] Sumimoto H., Kamakura S., Ito T. (2007). Structure and function of the PB1 domain, a protein interaction module conserved in animals, fungi, amoebas, and plants. Sci. STKE.

[14] Suzuki A., Ohno S. (2006). The PAR-aPKC system: Lessons in polarity. J. Cell Sci..

[15] Iden S., Collard J.G. (2008). Crosstalk between small GTPases and polarity proteins in cell polarization. Nat. Rev. Mol. Cell Biol..

[16] Hayase J., Kamakura S., Iwakiri Y., Yamaguchi Y., Izaki T., Ito T., Sumimoto H. (2013). The WD40 protein Morg1 facilitates Par6–aPKC binding to Crb3 for apical identity in epithelial cells. J. Cell Biol..

[17] Plant P.J., Fawcett J.P., Lin D.C.C., Holdorf A.D., Binns K., Kulkarni S., Pawson T. (2003). A polarity complex of mPar-6 and atypical PKC binds, phosphorylates and regulates mammalian Lgl. Nat. Cell Biol..

[18] Yamanaka T., Horikoshi Y., Sugiyama Y., Ishiyama C., Suzuki A., Hirose T., Iwamatsu A., Shinohara A., Ohno S. (2003). Mammalian Lgl forms a protein complex with PAR-6 and aPKC independently of PAR-3 to regulate epithelial cell polarity. Curr. Biol..

[19] Yamanaka T., Horikoshi Y., Izumi N., Suzuki A., Mizuno K., Ohno S. (2006). Lgl mediates apical domain disassembly by suppressing the PAR-3-aPKC-PAR-6 complex to orient apical membrane polarity. J. Cell Sci..

[20] Choi J., Troyanovsky R.B., Indra I., Mitchell B.J., Troyanovsky S.M. (2019). Scribble, Erbin, and Lano redundantly regulate epithelial polarity and apical adhesion complex. J. Cell Biol..

[21] Chen X., Macara I.G. (2005). Par-3 controls tight junction assembly through the Rac exchange factor Tiam1. Nat. Cell Biol..

[22] Ooshio T., Fujita N., Yamada A., Sato T., Kitagawa Y., Okamoto R., Nakata S., Miki A., Irie K., Takai Y. (2007). Cooperative roles of Par-3 and afadin in the formation of adherens and tight junctions. J. Cell Sci..

[23] Cong W., Hirose T., Harita Y., Yamashita A., Mizuno K., Hirano H., Ohno S. (2010). ASPP2 regulates epithelial cell polarity through the PAR complex. Curr. Biol..

[24] Yu M., Yang S., Qiu Y., Chen G., Wang W., Xu C., Cai W., Sun L., Xiao W., Yang H. (2015). Par-3 modulates intestinal epithelial barrier function through regulating intracellular trafficking of occludin and myosin light chain phosphorylation. J. Gastroenterol..

[25] Horikoshi Y., Suzuki A., Yamanaka T., Sasaki K., Mizuno K., Sawada H., Yonemura S., Ohno S. (2009). Interaction between PAR-3 and the aPKC-PAR-6 complex is indispensable for apical domain development of epithelial cells. J. Cell Sci..

[26] Hao Y., Du Q., Chen X., Zheng Z., Balsbaugh J.L., Maitra S., Shabanowitz J., Hunt D.F., Macara I.G. (2010). Par3 controls epithelial spindle orientation by aPKC-mediated phosphorylation of apical Pins. Curr. Biol..

[27] Durgan J., Kaji N., Jin D., Hall A. (2011). Par6B and atypical PKC regulate mitotic spindle orientation during epithelial morphogenesis. J. Biol. Chem..

[28] Linch M., Sanz-Garcia M., Soriano E., Zhang Y., Riou P., Rosse C., Cameron A., Knowles P., Purkiss A., Kjaer S., McDonald N.Q., Parker P.J. (2013). A cancer-associated mutation in atypical protein kinase Cι occurs in a substrate-specific recruitment motif. Sci. Signal..

[29] Fu D., Wakabayashi Y., Ido Y., Lippincott-Schwartz J., Arias I.M. (2010). Regulation of bile canalicular network formation and maintenance by AMP-activated protein kinase and LKB1. J. Cell Sci..

[30] Slim C.L., Lázaro-Diéguez F., Bijlard M., Toussaint M.J.M., de Bruin A., Du Q., Müsch A., van Ijzendoorn S.C.D. (2013). Par1b induces asymmetric inheritance of plasma membrane domains *via* LGN-dependent mitotic spindle orientation in proliferating hepatocytes. PLoS Biol..

[31] Homolya L., Fu D., Sengupta P., Jarnik M., Gillet J.-P., Vitale-Cross L., Gutkind J.S., Lippincott-Schwartz J., Arias I.M. (2014). LKB1/AMPK and PKA control ABCB11 trafficking and polarization in hepatocytes. PLoS One.

[32] Porat-Shliom N., Tietgens A.J., Itallie C.M., Vitale-Cross L., Jarnik M., Harding O.J., Anderson J.M., Gutkind J.S., Weigert R., Arias I.M. (2016). Liver kinase B1 regulates hepatocellular tight junction distribution and function *in vivo*. Hepatology.

[33] Izumi Y., Hirose T., Tamai Y., Hirai S., Nagashima Y., Fujimoto T., Tabuse Y., Kemphues K.J., Ohno S. (1998). An atypical PKC directly associates and colocalizes at the epithelial tight junction with ASIP, a mammalian homologue of *Caenorhabditis elegans* polarity protein PAR-3. J. Cell Biol..

[34] Takaki Y., Hirai S., Manabe N., Izumi Y., Hirose T., Nakaya M., Suzuki A., Mizuno K., Akimoto K., Tsukita S., Shuin T., Ohno S. (2001). Dynamic changes in protein components of the tight junction during liver regeneration. Cell Tissue Res..

[35] Wang T., Yanger K., Stanger B.Z., Cassio D., Bi E. (2014). Cytokinesis defines a spatial landmark for hepatocyte polarization and apical lumen formation. J. Cell Sci..

[36] Yuan H., Zhang H., Wu X., Zhang Z., Du D., Zhou W., Zhou S., Brakebusch C., Chen Z. (2009). Hepatocyte-specific deletion of Cdc42 results in delayed liver regeneration after partial hepatectomy in mice. Hepatology.

[37] Margolis B. (2018). The Crumbs3 polarity protein. Cold Spring Harb. Perspect. Biol..

[38] Lin D., Edwards A.S., Fawcett J.P., Mbamalu G., Scott J.D., Pawson T. (2000). A mammalian PAR-3–PAR-6 complex implicated in Cdc42/Rac1 and aPKC signalling and cell polarity. Nat. Cell Biol..

[39] Tamehiro N., Mujawar Z., Zhou S., Zhuang D.Z., Hornemann T., von Eckardstein A., Fitzgerald M.L. (2009). Cell polarity factor Par3 binds SPTLC1 and modulates monocyte serine palmitoyltransferase activity and chemotaxis. J. Biol. Chem..

[40] Lu M., Wu J., Hao Z.-W., Shang Y.-K., Xu J., Nan G., Li X., Chen Z.-N., Bian H. (2018). Basolateral CD147 induces hepatocyte polarity loss by E-cadherin ubiquitination and degradation in hepatocellular carcinoma progress. Hepatology.

[41] Sripathy S., Lee M., Vasioukhin V. (2011). Mammalian *Llgl2* is necessary for proper branching morphogenesis during placental development. Mol. Cell. Biol..

[42] Stevenson B.R., Siliciano J.D., Mooseker M.S., Goodenough D.A. (1986). Identification of ZO-1: A high molecular weight polypeptide associated with the tight junction (zonula occludens) in a variety of epithelia. J. Cell Biol..

[43] Otani T., Nguyen T.P., Tokuda S., Sugihara K., Sugawara T., Furuse K., Miura T., Ebnet K., Furuse M. (2019). Claudins and JAM-A coordinately regulate tight junction formation and epithelial polarity. J. Cell Biol..

[44] Joberty G., Petersen C., Gao L., Macara I.G. (2000). The cell-polarity protein Par6 links Par3 and atypical protein kinase C to Cdc42. Nat. Cell Biol..

[45] Jaffe A.B., Kaji N., Durgan J., Hall A. (2008). Cdc42 controls spindle orientation to position the apical surface during epithelial morphogenesis. J. Cell Biol..

[46] Lin R., Bagrodia S., Cerione R., Manor D. (1997). A novel Cdc42Hs mutant induces cellular transformation. Curr. Biol..

[47] Bryant D.M., Datta A., Rodríguez-Fraticelli A.E., Peränen J., Martín-Belmonte F., Mostov K.E. (2010). A molecular network for *de novo* generation of the apical surface and lumen. Nat. Cell Biol..

[48] Wojtal K.A., Diskar M., Herberg F.W., Hoekstra D., van IJzendoorn S.C.D. (2009). Regulatory subunit I-controlled protein kinase A activity is required for apical bile canalicular lumen development in hepatocytes. J. Biol. Chem..

[49] Fu D., Wakabayashi Y., Lippincott-Schwartz J., Arias I.M. (2011). Bile acid stimulates hepatocyte polarization through a cAMP-Epac-MEK-LKB1-AMPK pathway. Proc. Natl. Acad. Sci. U. S. A..

[50] Noda Y., Kohjima M., Izaki T., Ota K., Yoshinaga S., Inagaki F., Ito T., Sumimoto H. (2003). Molecular recognition in dimerization between PB1 domains. J. Biol. Chem..

[51] Almagor L., Ufimtsev I.S., Ayer A., Li J., Weis W.I. (2019). Structural insights into the aPKC regulatory switch mechanism of the human cell polarity protein lethal giant larvae 2. Proc. Natl. Acad. Sci. U. S. A..

[52] Müsch A., Cohen D., Yeaman C., Nelson W.J., Rodriguez-Boulan E., Brennwald P.J. (2002). Mammalian homolog of Drosophila tumor suppressor lethal (2) giant larvae interacts with basolateral exocytic machinery in Madin-Darby canine kidney cells. Mol. Biol. Cell.

[53] Dong W., Zhang X., Liu W., Chen Y., Huang J., Austin E., Celotto A.M., Jiang W.Z., Palladino M.J., Jiang Y., Hammond G.R.V., Hong Y. (2015). A conserved polybasic domain mediates plasma membrane targeting of Lgl and its regulation by hypoxia. J. Cell Biol..

[54] Bailey M.J., Prehoda K.E. (2015). Establishment of Par-polarized cortical domains *via* phosphoregulated membrane motifs. Dev. Cell.

[55] Holly R.W., Prehoda K.E. (2019). Phosphorylation of Par-3 by atypical protein kinase C and competition between its substrates. Dev. Cell.

[56] Renschler F.A., Bruekner S.R., Salomon P.L., Mukherjee A., Kullmann L., Schütz-Stoffregen M.C., Henzler C., Pawson T., Krahn M.P., Wiesner S. (2018). Structural basis for the interaction between the cell polarity proteins Par3 and Par6. Sci. Signal..

[57] Holly R.W., Jones K., Prehoda K.E. (2020). A conserved PDZ-binding motif in aPKC interacts with Par-3 and mediates cortical polarity. Curr. Biol..

[58] Zhang Y., De Mets R., Monzel C., Acharya V., Toh P., Chin J.F.L., Van Hul N., Ng I.C., Yu H., Ng S.S., Tamir Rashid S., Viasnoff V. (2020). Biomimetic niches reveal the minimal cues to trigger apical lumen formation in single hepatocytes. Nat. Mater..

[59] Takebe T., Sekine K., Enomura M., Koike H., Kimura M., Ogaeri T., Zhang R.R., Ueno Y., Zheng Y.W., Koike N., Aoyama S., Adachi Y., Taniguchi H. (2013). Vascularized and functional human liver from an iPSC-derived organ bud transplant. Nature.

[60] Low S.H., Wong S.H., Tang B.L., Subramaniam V.N., Hong W.J. (1991). Apical cell surface expression of rat dipeptidyl peptidase IV in transfected Madin-Darby canine kidney cells. J. Biol. Chem..

[61] Casanova J.E., Mishumi Y., Ikehara Y., Hubbard A.L., Mostov K.E. (1991). Direct apical sorting of rat liver dipeptidylpeptidase IV expressed in Madin-Darby canine kidney cells. J. Biol. Chem..

[62] Meder D., Shevchenko A., Simons K., Füllekrug J. (2005). Gp135/podocalyxin and NHERF-2 participate in the formation of a preapical domain during polarization of MDCK cells. J. Cell Biol..

[63] Russ A., Louderbough J.M.V., Zarnescu D., Schroeder J.A. (2012). Hugl1 and Hugl2 in mammary epithelial cells: Polarity, proliferation, and differentiation. PLoS One.

[64] Zihni C., Balda M.S., Matter K. (2014). Signalling at tight junctions during epithelial differentiation and microbial pathogenesis. J. Cell Sci..

[65] Rodriguez J., Peglion F., Martin J., Hubatsch L., Reich J., Hirani N., Gubieda A.G., Roffey J., Fernandes A.R., St Johnston D., Ahringer J., Goehring N.W. (2017). aPKC cycles between functionally distinct PAR protein assemblies to drive cell polarity. Dev. Cell.

[66] Nagai-Tamai Y., Mizuno K., Hirose T., Suzuki A., Ohno S. (2002). Regulated protein-protein interaction between aPKC and PAR-3 plays an essential role in the polarization of epithelial cells. Genes Cells.

[67] Kohjima M., Noda Y., Takeya R., Saito N., Takeuchi K., Sumimoto H. (2002). PAR3β, a novel homologue of the cell polarity protein PAR3, localizes to tight junctions. Biochem. Biophys. Res. Commun..

[68] Li D., Kuehn E.W., Prekeris R. (2014). Kinesin-2 mediates apical endosome transport during epithelial lumen formation. Cell. Logist..

[69] Klinkert K., Rocancourt M., Houdusse A., Echard A. (2016). Rab35 GTPase couples cell division with initiation of epithelial apico-basal polarity and lumen opening. Nat. Commun..

[70] Mangan A.J., Sietsema D.V., Li D., Moore J.K., Citi S., Prekeris R. (2016). Cingulin and actin mediate midbody-dependent apical lumen formation during polarization of epithelial cells. Nat. Commun..

[71] Konno T., Kohno T., Kikuchi S., Shimada H., Satohisa S., Takano K., Saito T., Kojima T. (2020). Localization of tricellular tight junction molecule LSR at midbody and centrosome during cytokinesis in human epithelial cells. J. Histochem. Cytochem..

[72] Takeuchi K., Sato N., Kasahara H., Funayama N., Nagafuchi A., Yonemura S., Tsukita S., Tsukita S. (1994). Perturbation of cell adhesion and microvilli formation by antisense oligonucleotides to ERM family members. J. Cell Biol..

[73] Noda Y., Takeya R., Ohno S., Naito S., Ito T., Sumimoto H. (2001). Human homologues of the *Caenorhabditis elegans* cell polarity protein PAR6 as an adaptor that links the small GTPases Rac and Cdc42 to atypical protein kinase C: Human PAR6s link Rac and Cdc42 to aPKC. Genes Cells.

[74] Mizushima S., Nagata S. (1990). pEF-BOS, a powerful mammalian expression vector. Nucleic Acids Res..

[75] Sekiya S., Suzuki A. (2011). Direct conversion of mouse fibroblasts to hepatocyte-like cells by defined factors. Nature.

[76] Matsumoto M., Matsuzaki F., Oshikawa K., Goshima N., Mori M., Kawamura Y., Ogawa K., Fukuda E., Nakatsumi H., Natsume T., Fukui K., Horimoto K., Nagashima T., Funayama R., Nakayama K. (2017). A large-scale targeted proteomics assay resource based on an *in vitro* human proteome. Nat. Methods.

